# Synthesis and characterization of bi(metallacycloprop-1-ene) complexes†

**DOI:** 10.1039/d2sc05378k

**Published:** 2022-11-16

**Authors:** Wei Bai, Long Yiu Tsang, Yilun Wang, Yang Li, Herman H. Y. Sung, Ian D. Williams, Guochen Jia

**Affiliations:** Department of Chemistry, The Hong Kong University of Science and Technology Clear Water Bay Kowloon Hong Kong P. R. China chjiag@ust.hk chwill@ust.hk; State Key Laboratory of Fine Chemicals, Department of Chemistry, School of Chemical Engineering, Dalian University of Technology Liaoning 116024 P. R. China chyangli@dlut.edu.cn; School of Chemical Engineering, Dalian University of Technology Panjin Liaoning 124221 P. R. China

## Abstract

In all previously reported metallacycloprop-1-ene or η^2^-vinyl complexes, the metal center bears only one vinyl moiety. We have now successfully synthesized and structurally characterized the first complexes bearing two η^2^-vinyl moieties or spiro bi(metallacycloprop-1-ene) complexes from reactions of alkynes with rhenium phosphine complexes. Computational studies indicate that the metallacycloprop-1-ene rings are aromatic and the complexes represent a rare σ-type spirometalla-aromatic system.

## Abstract 2

Well-defined metallacycloprop-1-ene complexes have only previously been isolated with a metal center bearing one vinyl moiety. We have successfully obtained the first examples of structurally characterized spiro bi(metallacycloprop-1-ene) complexes.

## Graphical abstract

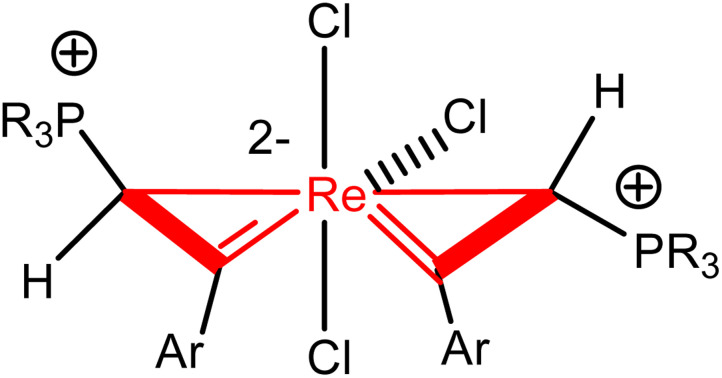

## Introduction

**Scheme 1:**
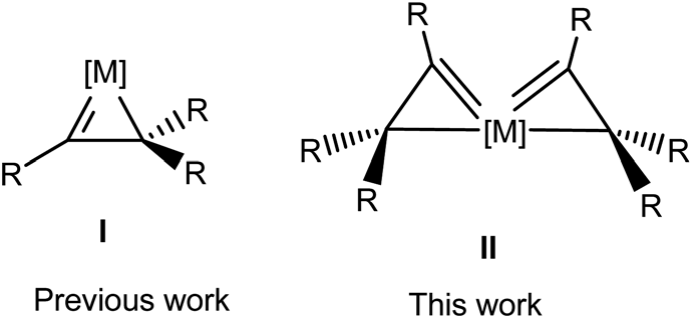
Structures of metallacycloprop-1-ene (I) and spiro bi[metallacycloprop-1-ene] (II) complexes.

Metallacycloprop-1-enes or η^2^-vinyl complexes (I, Scheme 1) are compounds with an organic vinyl fragment CR=CR_2_ bound asymmetrically to a metal center through both carbon atoms.^1^ As the smallest metalla-carbocycles with an M=C and an M–C bond, these complexes are receiving increasing attention for their roles in organometallic synthesis^2–9^ and catalysis,^10–16^ as well as for their aromatic properties.^17–20^

Well-defined metallacycloprop-1-ene complexes have been isolated with metals such as tungsten,^21^ molybdenum,^22^ rhenium,^23^ ruthenium,^24^ and osmium.^25^ In all these complexes, the metal center bears only one vinyl moiety (I, Scheme 1). In principle, a transition metal fragment may bind two or more η^2^-vinyl moieties to give interesting spiro metallacycloprop-1-ene complexes. However, such a possibility has not been previously demonstrated. In this work, we report the synthesis and characterization of the first complexes bearing two η^2^-vinyl moieties or spiro bi(metallacycloprop-1-ene) complexes (II, Scheme 1), a unique σ-type spiro metallaaromatic system.

## Results and discussion

**Scheme 2:**
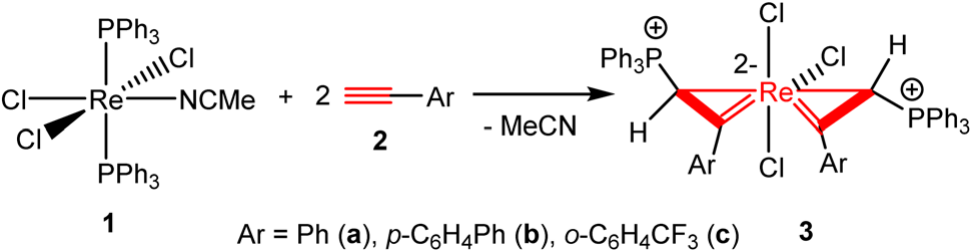
Syntheses of spiro bi(metallacycloprop-1-ene) complexes 3a–c.

The first spiro bi(metallacycloprop-1-ene) complex was obtained by the reaction of ReCl_3_(PPh_3_)_2_(MeCN) (1) with PhC≡CH. Heating a mixture of 1 and four equivalents of phenylacetylene (2a) in dichloromethane at 70 °C for 1 h produced a yellow solution, from which the complex Re{η^2^-C(Ph)=CH(PPh_3_)}_2_Cl_3_ (3a) can be isolated as an orange solid in 68.1% yield (Scheme 2). The analogous complexes Re{η^2^-C(Ar)=CH(PPh_3_)}_2_Cl_3_ (Ar = *p*-C_6_H_4_Ph (3b), *o*-C_6_H_4_CF_3_ (3c)) were obtained from the reactions of 1 with the corresponding aryl alkynes HC≡CAr under similar conditions. *In situ* NMR experiments indicate that 1 is also reactive with alkyl alkynes such as 1-pentyne and 1-ethynylcyclohexene. The reactions gave mixtures of unidentified species, and the expected bi(metallacycloprop-1-ene) complexes analogous to 3 were not produced.

**Fig. 1:**
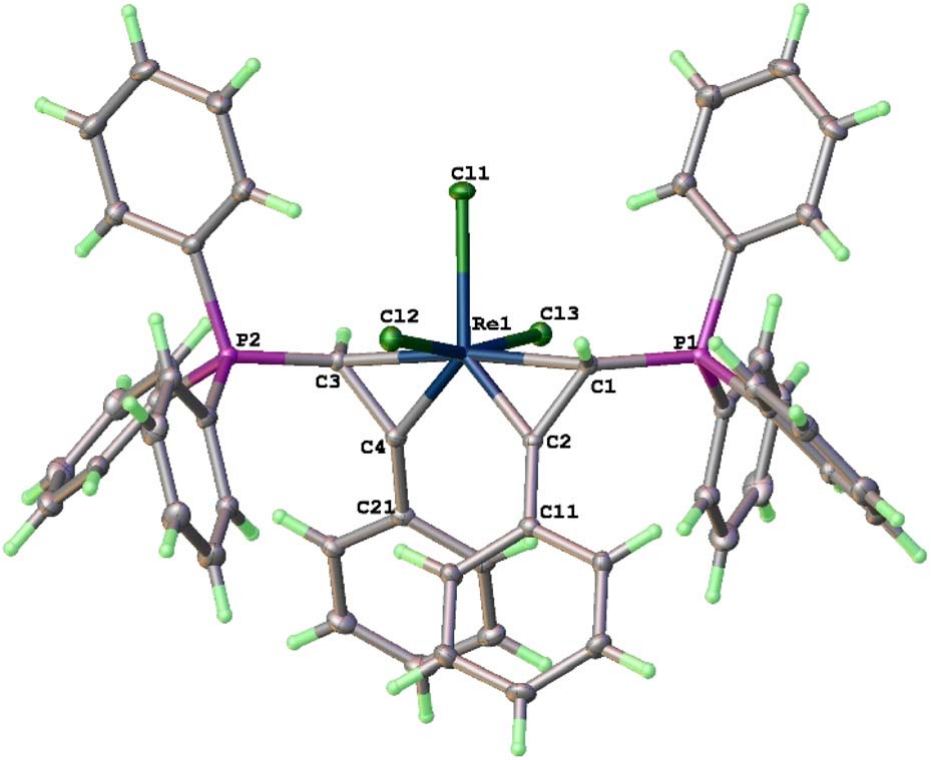
X-ray crystal structure of 3a (ellipsoids at the 40% probability level). Selected bond lengths [Å] and angles [°]: Re(1)–Cl(1) 2.4837(7), Re(1)–Cl(2) 2.4079(7), Re(1)–Cl(3) 2.4035(7), Re(1)–C(1) 2.211(3), Re(1)–C(2) 1.903(3), Re(1)–C(3) 2.205(3), Re(1)–C(4) 1.903(3), C(1)–C(2) 1.467(4), C(3)–C(4) 1.469(4), Cl(1)–Re(1)–Cl(2) 84.62(3), Cl(1)–Re–Cl(3) 82.80(2), Cl(2)–Re(1)–Cl(3) 167.30(2), C(1)–Re(1)–C(3) 172.65(10), C(1)–Re(1)–C(2) 40.92(11), C(1)–Re(1)–Cl(1), 95.18(8), C(2)–Re–C(4) 93.19(12), C(3)–Re(1)–C(4) 41.07(11), C(3)–Re(1)–Cl(1) 92.14(8).

The structure of 3a has been determined by X-ray diffraction. As shown in Fig. 1, complex 3a contains three meridionally bound Cl ligands and two η^2^-PhC=CH(PPh_3_) moieties. The Re–C(1) (2.211(3) Å) and Re–C(3) (2.205(3) Å) bond distances are typical of Re–C(sp^3^) bonds, while those of Re–C(2) and Re–C(4) (both are 1.903(3) Å) are typical of Re=C alkylidene double bonds. The C(1)–C(2) (1.467(4) Å) and C(3)–C(4) (1.469(4) Å) are typical of C–C single bonds with some associated π-character. The structural features associated with the Re(η^2^-CH(PPh_3_)CPh) fragments are similar to those of reported rhenacycloprop-1-ene complexes.^23^ Thus, complex 3a can be described as a spiro bi(metallacycloprop-1-ene) complex. The two spiro-connected rhenacycles are nearly co-planar or slightly twisted as evidenced by the twist angle between the MCC planes (25°). The two formal Re=C bonds are orthogonal *cis* to each other (93.19(12)°), while the two Re–C(sp^3^) single bonds are *trans* to each other (172.65(10)°). The two PPh_3_ substituents are oriented *trans* to each other. The spiro-concept derives from the conformational requirement of coplanarity of the aryl substituent with the metallacycloprop-1-ene ring, which twists either the C11 or C21 phenyl to the front. Both conformations are found in the crystal.

The presence of the Re{η^2^-C(Ar)=CH(PPh_3_)}_2_ moieties in 3 is supported by the NMR data. For example, the ^1^H NMR spectrum of 3b showed the C*H*(P) signal at 5.11 ppm. The ^31^P{^1^H} NMR spectrum of 3b showed a singlet at 24.5 ppm. The ^13^C{^1^H} NMR spectrum of 3b showed the Re=*C* signal at 251.4 ppm and the *C*H(P) signal at 21.3 ppm.

In the solid state, complexes 3 are air-stable and can be stored at room temperature for at least 6 months without appreciable decomposition. In solution, they are also air-stable for at least 3 hours. However, decomposition was noted when a solution of complexes 3 in a halogenated solvent was exposed to air for a day.

Complexes 3a–c are interesting as they represent the first examples of spiro bi(metallacycloprop-1-ene) complexes or complexes bearing two η^2^-vinyl ligands at the same metal center. Complexes 3a–c are also unique mononuclear rhenium complexes with two hydrocarbyl carbene ligands. It is noted that mononuclear complexes with two hydrocarbyl carbene ligands (CHR and CR_2_) are rare and limited to a few of those of Os,^26^ W,^27^ Mo,^28^ Nb,^29^ and Ta.^30^

**Scheme 3:**
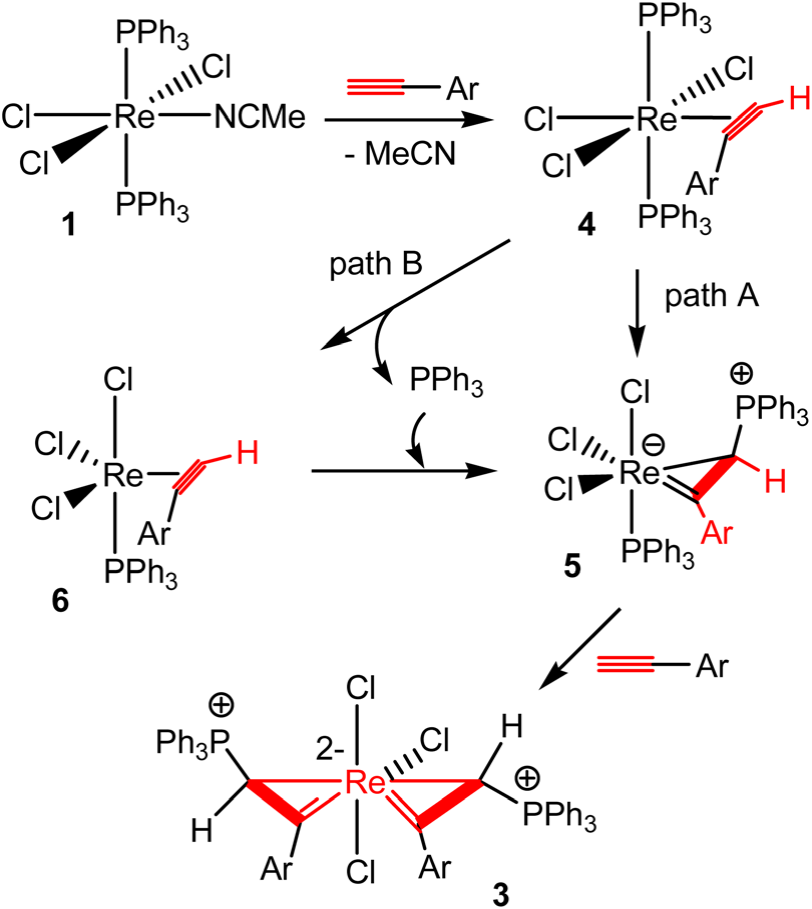
Proposed pathways for the formation of complexes 3.

Scheme 3 shows a plausible mechanism for the formation of spiro bi(metallacycloprop-1-ene) complexes 3 from the reactions of 1 with HC≡CAr. The MeCN ligand in 1 is labile. Thus, complex 1 could initially undergo a ligand substitution reaction with HC≡CAr to give an η^2^-alkyne complex ReCl_3_(η^2^-alkyne)(PPh_3_)_2_ (4). Complex 4 could evolve to the metallacycloprop-1-ene intermediate 5*via* the addition of a PPh_3_ to the terminal carbon of the coordinated aryl alkyne. Further reaction of intermediate 5 with another molecule of ArC≡CH would give a spiro bi(metallacycloprop-1-ene) complex 3. Both intramolecular migration of PR_3_ from metal to a coordinated alkyne^31^ and intermolecular attack of free PR_3_ on coordinated alkyne ligand^32^ have been suggested previously for addition reactions of phosphines with alkynes. Similar reaction pathways could be proposed for the addition of PPh_3_ to the ArC≡CH in the present case. For example, the intermediate 5 could be formed by intramolecular migration of a PPh_3_ ligand of rhenium in 4 to the terminal carbon of the coordinated aryl alkyne (path A), or dissociation of a PPh_3_ to give the complex 6, followed by intermolecular nucleophilic attack of the dissociated PPh_3_ at the terminal alkyne carbon in 6 (path B).

**Scheme 4:**
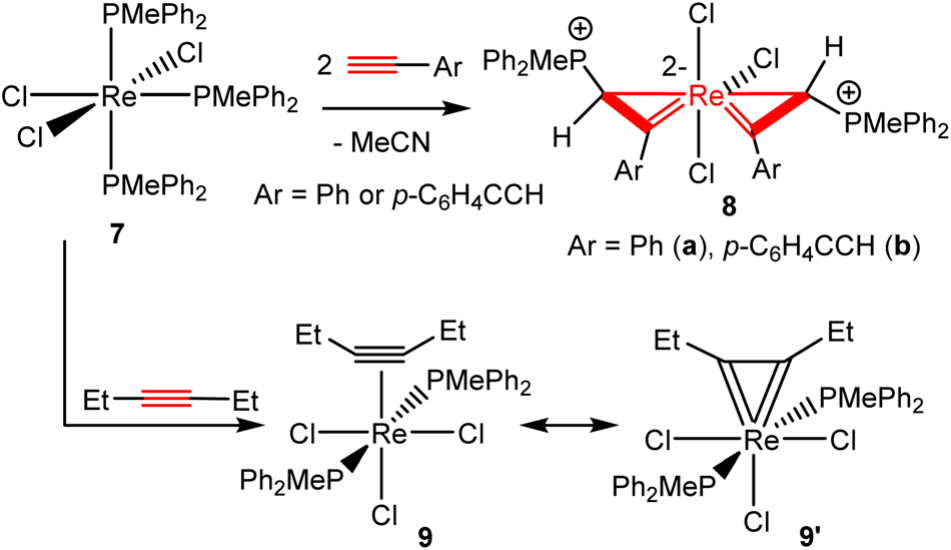
Reactions of complex 7 with alkynes.

Analogous spiro bi(metallacycloprop-1-ene) complexes could be obtained from reactions of terminal alkynes with other Re(iii) phosphine complexes. For example, the tris-phosphine complex ReCl_3_(PMePh_2_)_3_ (7) reacted with phenylacetylene (2a) and *p*-HC≡C–C_6_H_4_–C≡CH in toluene at 100 °C for 2 h to produce the corresponding spiro bi(metallacycloprop-1-ene) complexes 8a and 8b, respectively (Scheme 4). Like complexes 3, complexes 8 are also air-stable in the solid state. Preliminary experiments showed that 7 might also react with alkyl alkynes to give metallacycloprop-1-ene complexes. However, further work is needed to define the structures of the products.

**Fig. 2:**
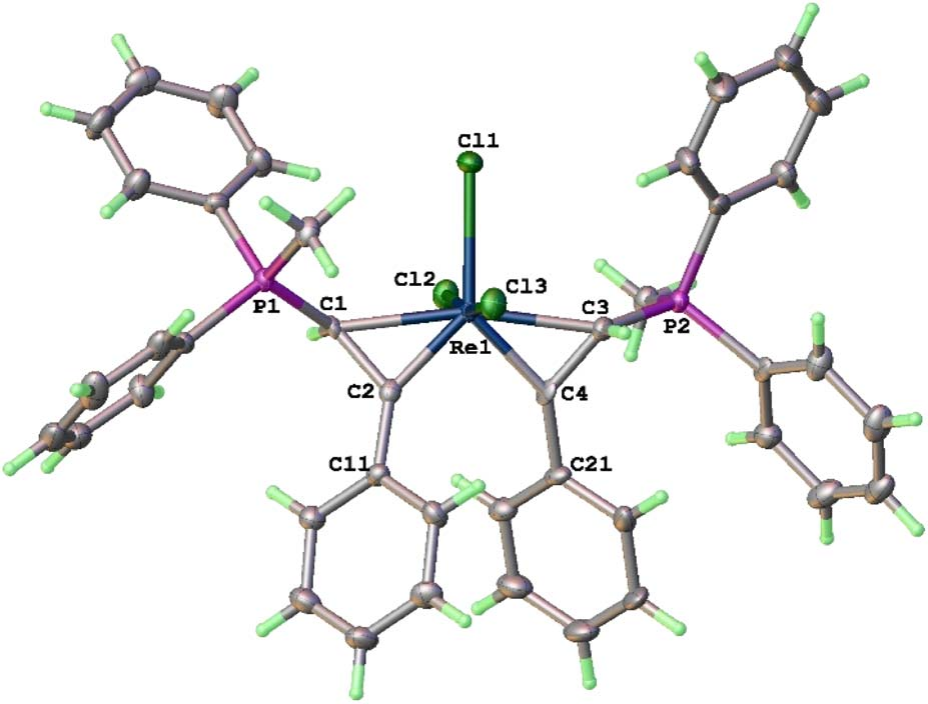
X-ray crystal structure of 8a (ellipsoids at the 40% probability level). Selected bond lengths [Å] and angles [°]: Re(1)–Cl(2) 2.420(3), Re(1)–Cl(1) 2.484(3), Re(1)–Cl(3) 2.430(2), Re(1)–C(1) 2.254(7), Re(1)–C(2) 1.885(9), Re(1)–C(3) 2.242(6), Re(1)–C(4) 1.873(7), C(1)–C(2) 1.455(10), C(3)–C(4) 1.437(14), C(1)–P(1) 1.748(8), C(3)–P(2) 1.765(8), Cl(2)–Re(1)–Cl(1) 85.29(7), Cl(2)–Re(1)–Cl(3) 170.24(9), C(1)–Re(1)–C(3) 165.8(3), C(1)–Re(1)–C(2) 39.9(3), C(1)–Re(1)–Cl(1) 97.3(2), C(2)–Re(1)–C(4) 90.0(3), C(3)–Re(1)–C(4) 39.6(4), C(3)–Re(1)–Cl(1) 96.9(3), Re(1)–C(1)–C(2) 56.2(4), Re(1)–C(3)–C(4) 56.2(3).

The structure of 8a has also been determined by X-ray diffraction (Fig. 2). In general, complex 8a has structural features similar to those of 3a. The twist angle between the two rhenacycles (30.5°) in 8a is slightly larger than that of 3a (25°). In agreement with the solid-state structure, the ^13^C{^1^H} NMR spectrum of 8a shows the Re=C signal at 254.5 ppm and that of Re–C*H*(P) at 11.3 ppm. The NMR data of 8b are similar to those of 8a (see ESI†).

**Scheme 5:**
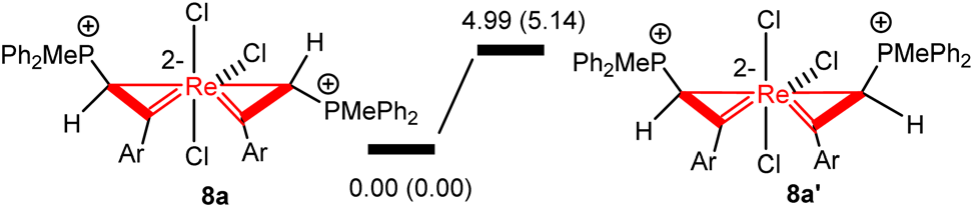
Relative energies of 8a and 8a′ isomers. The relative free energies and electronic energies (in parentheses) are given in kcal mol^−1^.

In the solid-state structures of both 3a and 8a, the two phosphonium substituents are oriented *trans* to each other. In principle, the two phosphonium substituents could also be oriented *cis* to each other. To understand the experimental observation, we optimized the structures of 8a (with *trans* disposed phosphonium substituents) and 8a′ (with *cis* disposed phosphonium substituents), and calculated their relative energies. As shown in Scheme 5, 8a is thermodynamically more stable than 8a′ by 4.99 kcal mol^−1^, consistent with the experimental observation that 8a is the only observed isomer.

We have also explored the possibility of the formation of bis(η^2^-vinyl) complexes by using internal alkynes. Heating a mixture of 7 and four equivalents of 3-hexyne in toluene at 100 °C for 2 h produced cleanly the alkyne complex Re(η^2^-EtC≡CEt)Cl_3_(PMePh_2_)_2_ (9), which can be isolated as a yellow solid in 89.9% yield (Scheme 4). The complex is diamagnetic and can be readily characterized by NMR spectroscopy. The ^31^P{^1^H} NMR in CDCl_3_ showed a singlet at −53.4 (s). The ^1^H NMR showed the Et signal at 0.91 (C*H*_3_) and 3.91 (C*H*_2_) ppm. The ^13^C{^1^H} NMR in CDCl_3_ showed the C≡C signal at 229.9 ppm. The ^13^C chemical shift of the alkyne signal (at 229.9 ppm) is characteristic of four-electron alkyne ligands.^33^

**Fig. 3:**
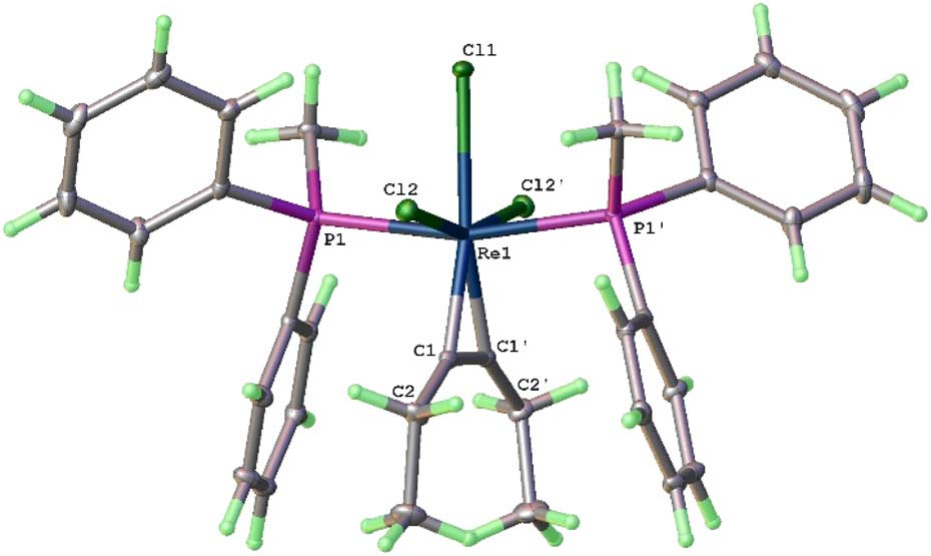
X-ray crystal structure of 9 (ellipsoids at the 40% probability level). Selected bond lengths [Å] and angles [°]: Re(1)–Cl(1) 2.4687(6), Re(1)–Cl(2) 2.4590(5), Re(1)–Cl(2^1^) 2.4591(5), Re(1)–C(1) 1.9642(19), Re(1)–C(1^1^) 1.9642(19), Re(1)–P(1) 2.4692(5), Re(1)–P(1^1^) 2.4692(5), C(1)–C(1^1^) 1.325(4), C(1)–C(2) 1.483(3), Cl(1)–Re(1)–Cl(2) 80.705(12), Cl(1)–Re(1)–Cl(2^1^) 80.704(11), Cl(2)–Re(1)–C(1) 79.63(6), C(1)–Re(1)–C(1^1^) 39.42(12), Re(1)–C(1)–C(1^1^) 70.29(6), Re(1)–C(1)–C(2) 142.52(16), P(1)–Re(1)–P(1^1^) 166.76(2).

The structure of 9 has been confirmed by X-ray diffraction. As shown in Fig. 3, it can be described as a distorted octahedral complex with two *trans*-disposed PMePh_2_ ligands at the axial positions, three meridionally bound Cl ligands, and an alkyne ligand in the equatorial position. Whereas structures 3a and 8a have 2-fold symmetry, complex 9 has crystallographic 2-fold symmetry, with the axis passing through Cl1 and Re1 and bisecting the coordinated 4e donor alkyne. The alkyne ligand is oriented perpendicular to the P(1)–Re–P(2) axis and bonded to rhenium symmetrically with the Re–C distance of 1.9642(19) Å, and the C≡C distance of the 1.325(4) Å. The structural parameters are similar to those of Cp*ReCl_2_(η^2^-EtC≡CEt) (Re–C, 1.961(3) and 1.969(3) Å; C≡C, 1.326(4) Å).^34^ The Re–C5 bond length (1.9642(19) Å) is within those reported for typical Re=CR_2_ (R=H or alkyl) carbene bonds (1.850–2.153 Å)^11*d*,14^ and shorter than those reported for Re–C bonds of typical rhenium–η^2^-alkyne (2e donor) complexes (2.118–2.247 Å).^35^ The NMR and the X-ray data suggest that this structure has contributions from the resonance forms 9 and 9′, with 9′ being dominant (Scheme 4). The successful isolation of 9 provides indirect evidence that complexes 3 and 8 are formed through the η^2^-alkyne complexes ReCl_3_(η^2^-alkyne)(PR_3_)_2_.

**Fig. 4:**
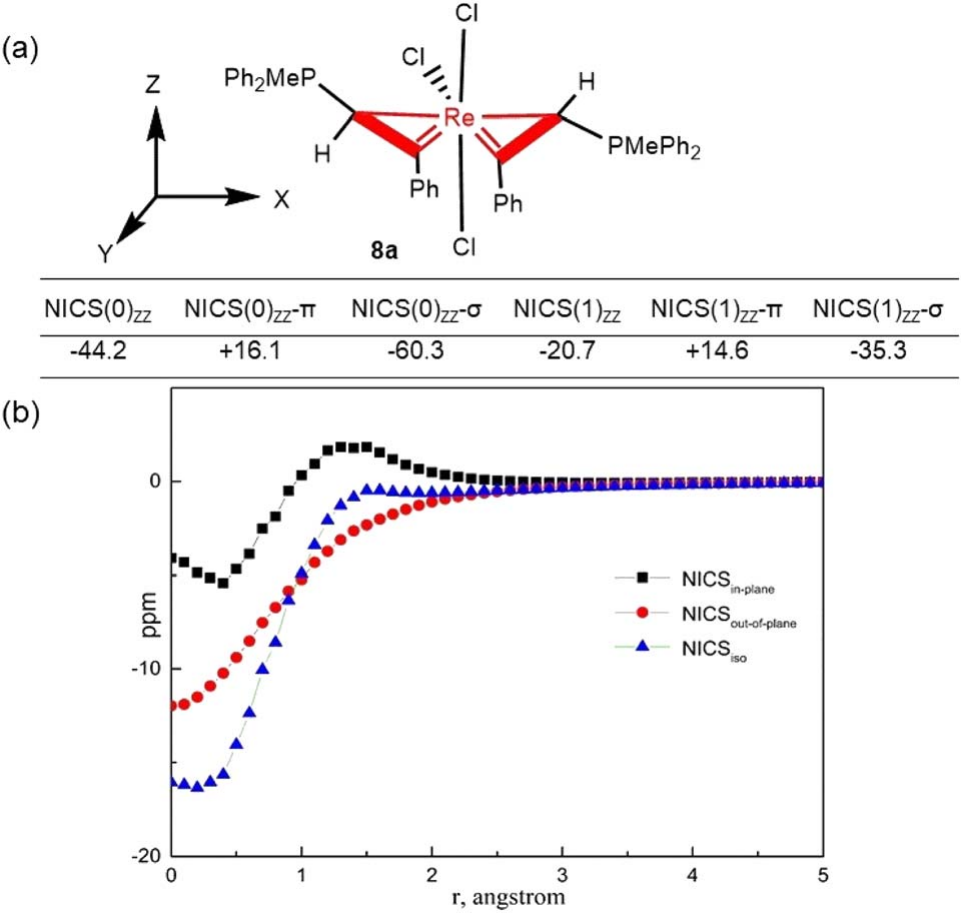
(a) Computed NICS values for complex 8a. (b) NICS-scan curves for 8a.

Cyclopropene^36,37^ and fused metallacycloprop-1-ene complexes^17–20,38^ have been shown to display σ-aromaticity. We expect that complexes 3 and 8 might also show aromatic character. To verify the hypothesis, we have used a series of theoretical methods to probe the aromatic properties of spiro bi(metallacycloprop-1-ene) complex 8a. We first calculated the values of nucleus-independent chemical shift (NICS), a common index for aromaticity.^39^ Since 8a has a pseudo 2-fold symmetric structure, the two three-membered rings are chemically equivalent. As shown in Fig. 4a, the NICS(0)_*ZZ*_ values and NICS(1)_*ZZ*_ values are −44.2 and −20.7 ppm, respectively, indicating that the metallacycle is aromatic. Canonical molecular orbital (CMO) NICS calculations reveal that the NICS(0)_*ZZ*_-π value from π molecular orbitals (+16.1 ppm) has a positive sign, while the NICS(0)_*ZZ*_-σ value from the key occupied σ molecular orbitals (−60.3 ppm) has a negative sign, suggesting the possible σ-aromaticity. Consistent with the σ-aromaticity, the NICS(1)_*ZZ*_-π value (+14.6 ppm) also has a positive sign, while the NICS(1)_*ZZ*_-σ value orbitals (−35.3 ppm) has a negative sign.

To verify the σ-aromaticity, complex 8a was studied by the NICS-scan^40^ and NICS_in–out_^41^ techniques, which can draw inferences about types of aromaticity based on the shape of the curves. To avoid the influence of the ligands in the structure, the NICS values of the metallacycle in 8a were decomposed into atomic contributions in the framework of the “atoms in molecules (AIM)” theory^42^ using the AIMAll^43^ program. As shown in Fig. 4b, the NICS out-of-plane curve does not exhibit a pronounced minimum at non-zero *r* values. The NICS in–out curve is not arc-shaped (Fig. S1, ESI†), as expected for typical π aromatic systems.^44^ For comparison, NICS-scan and NICS_in–out_ shapes without AIM processing are listed in Fig. S2 and S3.†

The anisotropy of the induced current density (AICD) analysis^45^ is another commonly used method for the evaluation of aromaticity. As shown in Fig. S4,† the σ-system of the three-membered metallacycles of 8a shows a diatropic ring current, confirming the σ-aromaticity. The σ-aromaticity of 8a is further elucidated by the gauge including magnetically induced currents (GIMIC).^46^ The GIMIC maps in the plane of a single ring of 8a are shown in Fig. S5,† which vividly display that significant diatropic currents formed in both the inner and outer edges of the three-membered metallacycle. To visualize the induced current of 8a, the induced current modulus surfaces were built up. The resulting Jmod plot (Fig. S6†) clearly shows that the diatropic surfaces (blue) around the single metallacycle are large and closed whereas the paratropic surfaces (red) inside the rings are significantly smaller. The static streamline plot of 8a (Fig. S7†) shows strong net currents circulating the single metallacycle cycle, corresponding to diamagnetic, *i.e.*, aromatic, current. Thus the GIMIC results, both Jmod and streamline plots, confirm the aromatic character of the metallacycle of 8a.

**Fig. 5:**
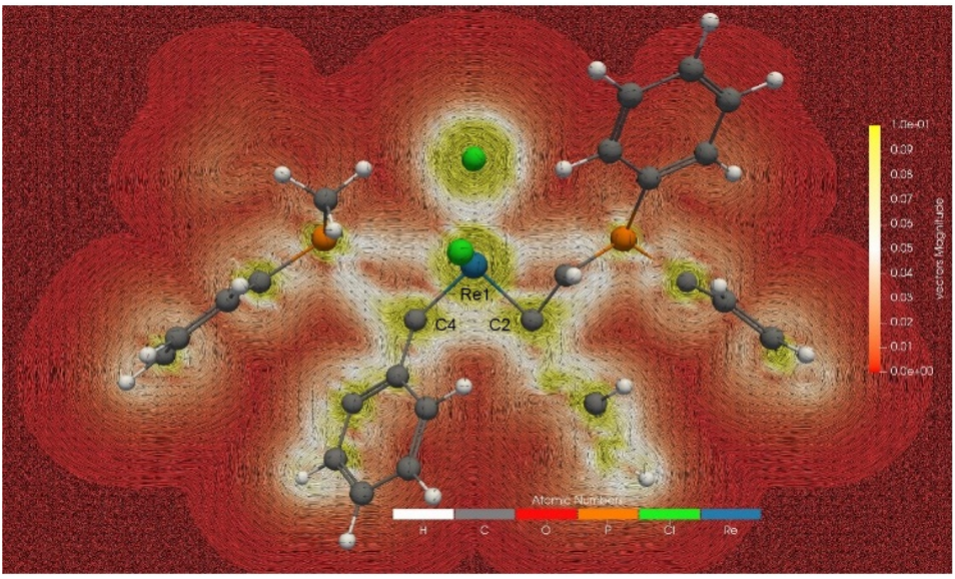
The GIMIC map of 8a in-plane formed by the three atoms of Re1, C2, and C4. The magnetic field vector is orthogonal concerning the ring plane and points upward (clockwise currents are diatropic).

Having confirmed the σ-aromaticity of mono-metallacycle, we have also investigated the global aromaticity of 8a.^47^ A global π-aromatic system has diatropic ring current circuiting along the outmost periphery. The GIMIC map with the magnetic field direction perpendicular to the plane formed by the three atoms of Re1, C2, and C4 (Fig. 5) shows induced currents generated in 8a connecting the two non-coplanar three-membered metallacycle rings *via* the unbound C2 and C4 atoms (the calculated C2–C4 Mayer bond order is only 0.05). The induced current between C2 and C4 in 8a is further proved by the current intensity through the integral plane calculated by the GIMIC program, a method that enables one to quantitatively check the aromaticity according to the induced current. As shown in Fig. S8,† the current intensity passing through the bonds of the three-membered ring is greater than 8.6 nA T^−1^, while that between C2 and C4 atoms is 7.04 nA T^−1^.

The magnetically induced current can be visualized *via* the AIMAll program. Fig. S9a and b† show that the magnetically induced current between the two non-coplanar three-membered metallacycle rings in 8a is connected to form an overall diatropic induced current. The current intensity between C2 and C4 atoms is slightly weaker than that on the ring plane, but it cannot be ignored. The NICS-grid (Fig. S9c†) and ICSS plots (Fig. S9d†) based on the NICS method also show that the two non-coplanar three-membered metallacycles of 8a form an overall shielded area. These results suggest that the two three-membered metallacycles in 8a constitute a special 3D global σ-aromaticity, with the induced current flowing *via* the unbound C2 and C4 atoms.

There has been much interest in the chemistry of metallaaromatics.^38,48^ Spiro metalla-aromatics, in which two or more aromatic rings are fused by sharing a single metal atom (the spiro atom), are novel metallaaromatic systems introduced recently. For example, Xi and coworkers have realized π-type bis-spirometalla-aromatics with square planar^49^ and tetrahedral^50^ geometries, and tris-spiroaromatics with a hexalithio spiro vanadacycle.^51^ Complexes 3 and 8 represent unique examples of σ-type spiro metallaaromatics.

## Conclusions

In summary, we have successfully obtained the first examples of spiro bi(metallacycloprop-1-ene) complexes from reactions of alkynes with rhenium phosphine complexes. Computational studies indicate that the metallacycloprop-1-ene rings are aromatic and thus the complexes represent a rare σ-type spirometallaaromatic system.

## Data availability

Crystallographic data for 3a (CCDC no. 2205733), 8a (CCDC no. 2205745), and 9 (CCDC no. 2205743) have been deposited at The Cambridge Crystallographic Data Centre.†

## Author contributions

G. J. conceived the project and supervised the findings of this work. W. B. and L. Y. T. carried out the syntheses and characterizations. Y. W. and Y. L. performed the computations. H. H. Y. S. and I. D. W. performed the XRD. G. J., W. B., I. D. W. and Y. W. wrote the manuscript and all authors contributed to the final manuscript.

## Conflicts of interest

There are no conflicts to declare.

## Supplementary Material

SC-014-D2SC05378K-s001

SC-014-D2SC05378K-s002

## References

[cit1] Frohnapfel D. S., Templeton J. L. (2000). Coord. Chem. Rev..

[cit2] Ehweiner M. A., Peschel L. M., Stix N., Ćorovic M. Z., Belaj F., Mösch-Zanetti N. C. (2021). Inorg. Chem..

[cit3] Curto S. G., Esteruelas M. A., Oliván M., Oñate E., Vélez A. (2018). Organometallics.

[cit4] Zhu C., Wu J., Li S., Yang Y., Zhu J., Lu X., Xia H. (2017). Angew. Chem., Int. Ed..

[cit5] Luo M., Sui Y., Lin X., Zhu C., Yan Z., Ruan Y., Zhang H., Xia H. (2020). Chem. Commun..

[cit6] Castro-Rodrigo R., Esteruelas M. A., López A. M., López F., Mascareñas J. L., Oliván M., Oñate E., Saya L., Villarino L. (2010). J. Am. Chem. Soc..

[cit7] Niikura F., Seino H., Mizobe Y. (2009). Organometallics.

[cit8] Kurogi T., Mindiola D. J. (2020). Organometallics.

[cit9] Li X., Vogel T., Incarvito C. D., Crabtree R. H. (2005). Organometallics.

[cit10] Galiana-Cameo M., Romeo R., Urriolabeitia A., Passarelli V., Pérez-Torrente J. J., Polo V., Castarlenas R. (2022). Angew. Chem., Int. Ed..

[cit11] Fürstner A. (2019). J. Am. Chem. Soc..

[cit12] Roşca D.-A., Radkowski K., Wolf L. M., Wagh M., Goddard R., Thiel W., Fürstner A. (2017). J. Am. Chem. Soc..

[cit13] Sánchez-Page B., Munarriz J., Jiménez M. V., Pérez-Torrente J. J., Blasco J., Subias G., Passarelli V., Álvarez P. (2020). ACS Catal..

[cit14] Zhang M., Huang G. (2016). Chem.–Eur. J..

[cit15] Imazaki Y., Shirakawa E., Ueno R., Hayashi T. (2012). J. Am. Chem. Soc..

[cit16] Jiang J., Liu H., Cao L., Zhao C., Liu Y., Ackermann L., Ke Z. (2019). ACS Catal..

[cit17] Huang Y., Dai C., Zhu J. (2020). Chem.–Asian J..

[cit18] Huang F., Zheng X., Lin X., Ding L., Zhuo Q., Wen T. B., Zhang H., Xia H. (2020). Chem. Sci..

[cit19] Bai W., Sun Y., Wang Y., Zhou Y., Zhao Y., Bao X., Li Y. (2022). Chem. Commun..

[cit20] Batuecas M., Castro-Rodrigo R., Esteruelas M. A., García-Yebra C., López A. M., Oñate E. (2016). Angew. Chem., Int. Ed..

[cit21] Stone K. C., Jamison G. M., White P. S., Templeton J. L. (2003). Organometallics.

[cit22] Buccella D., Janak K. E., Parkin G. (2008). J. Am. Chem. Soc..

[cit23] He G., Chen J., Sung H. H.-Y., Williams I. D., Jia G. (2021). Inorg. Chim. Acta.

[cit24] Bernal M. J., Torres O., Martin M., Sola E. (2013). J. Am. Chem. Soc..

[cit25] Álvarez-Pérez A., González-Rodríguez C., García-Yebra C., Varela J. A., Oñate E., Esteruelas M. A., Saá C. (2015). Angew. Chem., Int. Ed..

[cit26] Wang H.-X., Wan Q., Low K.-H., Zhou C.-Y., Huang J.-S., Zhang J.-L., Che C.-M. (2020). Chem. Sci..

[cit27] Benedikter M. J., Musso J. V., Frey W., Schowner R., Buchmeiser M. R. (2021). Angew. Chem., Int. Ed..

[cit28] Paul B., Schrock R. R., Tsay C. (2021). Organometallics.

[cit29] Kilgore U. J., Tomaszewski J., Fan H., Huffman J. C., Mindiola D. J. (2007). Organometallics.

[cit30] Diminnie J. B., Hall H. D., Xue Z. (1996). Chem. Commun..

[cit31] Kuniyasu H., Nakajima T., Tamaki T., Iwasaki T., Kambe N. (2015). Organometallics.

[cit32] Allen A., Lin W. (1999). Organometallics.

[cit33] Templeton J. L., Ward B. C. (1980). J. Am. Chem. Soc..

[cit34] Herrmann W. A., Fischer R. A., Herdtweck E. (1989). Organometallics.

[cit35] Kowalczyk J. J., Arif A. M., Gladysz J. A. (1991). Organometallics.

[cit36] Wu J., Liu X., Hao Y., Chen H., Su P., Wu W., Zhu J. (2018). Chem.–Asian J..

[cit37] Aysin R. R., Leites L. A., Bukalov S. S. (2020). Organometallics.

[cit38] Chen D., Hua Y., Xia H. (2020). Chem. Rev..

[cit39] Fallah-Bagher-Shaidaei H., Wannere C. S., Corminboeuf C., Puchta R., Schleyer P. v. R. (2006). Org. Lett..

[cit40] Stanger A. J. (2006). J. Org. Chem..

[cit41] Torres-Vega J. J., Vásquez-Espinal A., Caballero J., Valenzuela M. L., Alvarez-Thon L., Osorio E., Tiznado W. (2014). Inorg. Chem..

[cit42] (a) Bader R. F. W. , Atoms in Molecules: a Quantum Theory, Clarendon Press, Oxford, New York, 1990

[cit43] Keith T. A. , AIMAll, TK Gristmill Software, Overland Park, KS, USA, 2017

[cit44] Leites L. A., Bukalov S. S., Aysin R. R., Piskunov A. V., Chegerev M. G., Cherkasov V. K., Zabula A. V., West R. (2015). Organometallics.

[cit45] Geuenich D., Hess K., Köhler F., Herges R. (2005). Chem. Rev..

[cit46] Fliegl H., Taubert S., Lehtonen O., Sundholm D. (2011). Phys. Chem. Chem. Phys..

[cit47] Li Z., Hou X., Han Y., Fan W., Ni Y., Zhou Q., Zhu J., Wu S., Huang K.-W., Wu J. (2022). Angew. Chem., Int. Ed..

[cit48] Zhang Y., Yu C., Huang Z., Zhang W.-X., Ye S., Wei J., Xi Z. (2021). Acc. Chem. Res..

[cit49] Zhang Y., Wei J., Chi Y., Zhang X., Zhang W.-X., Xi Z. (2017). J. Am. Chem. Soc..

[cit50] Zhang Y., Wei J., Zhu M., Chi Y., Zhang W.-X., Ye S., Xi Z. (2019). Angew. Chem., Int. Ed..

[cit51] Huang Z., Zhang Y., Zhang W.-X., Wei J., Ye S., Xi Z. (2021). Nat. Commun..

