# Supplementary material for: Synthesis and characterization of bi(metallacycloprop-1-ene) complexes
Source: Chem Sci. 2022 Nov 16;14(1):96–102. doi: 10.1039/d2sc05378k (PMC9769101; doi:10.1039/d2sc05378k)
Supplement: SC-014-D2SC05378K-s001 [file SC-014-D2SC05378K-s001.pdf]

Electronic Supplementary Material (ESI) for Chemical Science.
This journal is © The Royal Society of Chemistry 2022

Synthesis and Characterization of Bi(metallacycloprop-1-ene) Complexes

Wei Bai,[a,b] Long Yiu Tsang,[a] Yilun Wang,[b,c] Yang Li,*[b,c] Herman H. Y. Sung,[a] Ian D. Williams*[a] and Guochen Jia*[a]

[a] Department of Chemistry, The Hong Kong University of Science and Technology, Clear Water Bay, Kowloon, Hong Kong, P.R. China

[b] State Key Laboratory of Fine Chemicals, Department of Chemistry, School of Chemical Engineering, Dalian University of Technology, Liaoning 116024, P.R. China

[c] School of Chemical Engineering, Dalian University of Technology, Panjin, Liaoning 124221, P.R. China

Supporting Information

Table of Contents

1. Experimental details **S2-S3**

2. Computational studies **S4-S9**

3. X-ray crystallographic study of complexes 3a, 8a and 9 **S10-S11**

4. NMR spectra **S12-S20**

5. The Calculated Cartesian Coordinates **S21-S25**

6. Reference **S26**

1. Experimental details

General information. All manipulations were carried out under a nitrogen atmosphere using standard Schlenk techniques unless otherwise stated. Solvents were distilled under nitrogen from sodium benzophenone (hexane, ether, tetrahydrofuran THF), sodium (toluene), or CaH_2 (dichloromethane, DCM). The starting materials $ReCl_3(PPh_3)_2(N{\equiv}CMe)$ [1] and $ReCl_3(PMePh_2)_3$ [2] were prepared following the procedure described in the literature. All other reagents were used as purchased from Aldrich Chemical Co.. Microanalyses were performed by M-H-W Laboratories (Phoenix, AZ) or MEDAC Ltd (Egham, UK). ^1H, $^{13}C\{^1H\}$, and $^{31}P\{^1H\}$ NMR spectra were collected on a Bruker-400 spectrometer (400 MHz). ^1H and ^{13}C NMR shifts are relative to TMS, and ^{31}P chemical shifts are relative to 85% H_3PO_4.

Preparation of Re(η²-C(Ph)CH(PPh₃))₂Cl₃ (3a). A mixture of $ReCl_3(PPh_3)_2(N{\equiv}CMe)$ (500 mg, 0.503 mmol) and phenylacetylene (0.116 mL, 1.06 mmol) in DCM (20 mL) was heated at 70 °C for 1 h in a sealed Schlenk tube. The reddish solution was transferred to a Schlenk flask and the solvent was removed vacuum to give an oily residue. The residue was treated with hexane (10 mL) to give an orange precipitate, which was collected by filtration. The solid was washed with acetone until the filtrate not showing a darken reddish colour. The remaining orange solid was dried under vacuum. Yield: 350 mg, 68.1%. $^{31}P\{^1H\}$ NMR (162 MHz, $CDCl_3$): δ 23.80. ^1H NMR (400 MHz, $CDCl_3$): δ 7.70 (m, 11H, *Ph*), 7.58 – 7.48 (m, 7H, *Ph*), 7.38 (m, 12H, *Ph*), 6.88 – 6.62 (m, 10H, Re=C*Ph*), 5.14 (d, J = 12.1 Hz, 2H, C*H*(PPh_3)). Anal. Calcd. for $C_{52}H_{42}Cl_3P_2Re \cdot 2(CH_2Cl_2)$: C, 54.45; H, 3.89. Found: C, 54.46; H, 3.85.

Preparation of Re(η²-C(4-PhC₆H₄)CH(PPh₃))₂Cl₃ (3b). A mixture of $ReCl_3(PPh_3)_2(N{\equiv}CMe)$ (100 mg, 0.117 mmol) and 4-ethynylbiphenyl (0.044 g, 0.247 mmol) in DCM (5 mL) was heated at 70 °C for 1 h in a sealed Schlenk tube. The reddish solution was transferred to a Schlenk flask and the solvent was removed vacuum to give an oily residue. The residue was treated with hexane (10 mL) to give an orange precipitate, which was collected by filtration. The solid was washed with acetone until the filtrate not showing a darken reddish colour. The remaining red-orange solid was dried under vacuum. Yield: 90 mg, 65.1%. $^{31}P\{^1H\}$ NMR (162 MHz, $CDCl_3$): δ 24.53. ^1H NMR (400 MHz, $CDCl_3$): δ 7.76 (m, 11H, *Ph*), 7.52 (m, 7H, *Ph*), 7.41 (m, 12H, *Ph*), 7.26 (m, 10H, C_6H_4-*Ph*), 6.94 (d, J = 8.0 Hz, 4H, C_6H_4), 6.78 (d, J = 8.0 Hz, 4H, C_6H_4), 5.11 (d, J = 12.2 Hz, 2H, C*H* (PPh_3)). $^{13}C\{^1H\}$ NMR (101 MHz, $CDCl_3$) : δ 251.51-251.37 (m, Re=*C*), 153.09 – 118.88 (m), 21.33 (d, ^1J(PC) = 69.3 Hz, *C*H(PPh_3)). Anal. Calcd. for $C_{64}H_{50}Cl_3P_2Re \cdot CH_2Cl_2$: C, 62.03; H, 4.16. Found: C, 61.46; H, 4.06.

Preparation of Re(η²-C(2-CF₃C₆H₄)CH(PPh₃))₂Cl₃ (3c). A mixture of $ReCl_3(PPh_3)_2(N{\equiv}CMe)$ (100 mg, 0.117 mmol) and 2-ethynyl-α,α,α-trifluorotoluene (0.0344 mL, 0.247 mmol) in DCM (5 mL) was heated at 70 °C for 1 h in a sealed Schlenk tube. The reddish solution was transferred to a Schlenk flask and the solvent was removed vacuum to give an oily residue. The residue was treated with hexane (10 mL) to give an orange precipitate, which was collected by filtration. The solid was washed with acetone until the filtrate not showing a darken reddish colour. The remaining yellow solid was dried under vacuum. Yield: 69 mg, 51.3%. $^{31}P\{^1H\}$ NMR (162 MHz, $CDCl_3$): δ 25.29. ^1H NMR (400 MHz, $CDCl_3$) δ 7.71 (t, 12H, *Ph*), 7.46 – 7.36 (m, 6H,

Ph), 7.29 (m, 12H, *Ph*), 6.94 – 6.86 (m, 4H), 6.68 (dt, *J* = 5.5, 2.0 Hz, 4H), 5.60 (d, *J* = 14.5 Hz, 2H). $^{13}C\{^1H\}$ NMR (101 MHz, $CDCl_3$): δ 254.11 – 253.96 (m, Re=*C*), 163.71 – 118.13 (m), 124.71 (d, ^1J(FC) = 5.0 Hz, *C*F_3), 21.91 (d, ^1J(PC) = 68.5 Hz, *C*H(PPh_3)). Anal. Calcd. for $C_{54}H_{40}Cl_3F_6P_2Re$: C, 56.04; H, 3.48. Found: C, 55.67; H, 3.34.

Preparation of Re(η²-C(Ph)CH($PMePh_2$))$_2$$Cl_3$ (8a). A mixture of $ReCl_3(PMePh_2)_3$ (200 mg, 0.224 mmol) and phenylacetylene (0.1 mL, 0.911 mmol) in toluene (10 mL) was heated at 100 °C for 2 h. The mixture was filtered and the yellow solid was washed with methanol (5 mL x 2) and ether (5 mL) to give Re(η²-C(Ph)=CH($PMePh_2$))$_2$$Cl_3$ (**8a**) as a yellow solid which was dried under vacuum. Yield: 175 mg, 87.1%. $^{31}P\{^1H\}$ NMR (162.0 MHz, $CDCl_3$): δ 25.2 (s). ^1H NMR (400.1 MHz, $CDCl_3$): δ 2.27 (d, ^2J(PH) = 14.0 Hz, 6 H, P*Me*Ph_2), 4.92 (d, ^2J(PH) = 11.2 Hz, 2 H, C*H*($PMePh_2$)), 6.58-6.74 (m, 10 H, *Ph*), 7.33-7.60 (m, 16 H, *Ph*), 8.23-8.27 (m, 4 H, *Ph*). $^{13}C\{^1H\}$ NMR (100.6 MHz, $CDCl_3$): δ 254.5 (d, ^2J(PC) = 15.7 Hz, Re=*C*), 144.2, 134.0-126.5, 21.9 (d, ^2J (PC) = 69.8 Hz, *C*H($PMePh_2$)), 21.8 (d, ^2J (PC) = 70.3 Hz, *C*H($PMePh_2$)), 11.3 (d, ^1J (PC) = 56.0 Hz, P*Me*Ph_2). Anal. Calcd. for $C_{42}H_{38}Cl_3P_2Re$: C, 56.22; H, 4.27. Found: C, 56.13; H,4.40.

Preparation of Re(η²-C(4-HC≡CC_6H_4)CH($PMePh_2$))$_2$$Cl_3$ (8b). A mixture of $ReCl_3(PMePh_2)_3$ (180 mg, 0.202 mmol) and 1,4-diethynylbenzene (56 mg, 0.444 mmol) in toluene (10 mL) was heated at 100 °C for 2 h. The mixture was loaded to a SiO_2 column and the eluted sequentially with DCM, and a mixture of DCM and ether (*v/v* = 10/1). The yellow band was collected and the solvents were removed under reduced pressure to give the complex **8b** as a brownish solid. Yield: 30 mg, 15.7%. $^{31}P\{^1H\}$ NMR (162.0 MHz, $CDCl_3$): δ 24.8 (s). ^1H NMR (400.1 MHz, $CDCl_3$): δ 2.28 (d, ^2J(PH) = 13.6 Hz, 6 H, P*Me*Ph_2), 2.99 (s, 2 H, C≡C*H*), 4.92 (d, ^2J(PH) = 11.2 Hz, 2 H, C*H*($PMePh_2$)), 6.52 (d, *J* = 8.4 Hz, 4 H, *C_6H_4*), 6.80 (d, *J* = 8.0 Hz, 4 H, *C_6H_4*), 7.33-7.60 (m, 16 H, *Ph*), 8.20-8.24 (m, 4 H, *Ph*). $^{13}C\{^1H\}$ NMR (100.6 MHz, $CDCl_3$): δ 252.6 (d, ^2J(PC) = 15.5 Hz, Re=*C*), 144.4, 134.0-126.2, 121.0, 84.2 (s, *C*≡CH), 78.3 (s, C≡*C*H), 22.9 (d, ^2J (PC) = 70.5 Hz, *C*H($PMePh_2$)), 22.8 (d, ^2J (PC) = 70.1 Hz, *C*H($PMePh_2$)), 11.4 (d, ^1J (PC) = 56.1 Hz, P*Me*Ph_2). Anal. Calcd. for $C_{46}H_{38}Cl_3P_2Re$: C, 58.45; H, 4.05. Found: C, 58.70; H,4.26.

Preparation of Re(η²-EtC≡CEt)Cl_3($PMePh_2$)$_2$ (9). A mixture of $ReCl_3(PMePh_2)_3$ (200 mg, 0.224 mmol) and 3-hexyne (0.1 mL, 0.880 mmol) in toluene (10 mL) was heated at 100 °C for 2 h. The volume of the yellow solution was reduced to ca. 2 mL and hexane (10 mL) was added to precipitate out a yellow solid. The yellow solid of Re(η²-EtC≡CEt)Cl_3($PMePh_2$)$_2$ (**9**) was washed with methanol (3 mL x 2) and ether (5 mL), then dried under vacuum. Yield: 156 mg, 89.9%. $^{31}P\{^1H\}$ NMR (162.0 MHz, $CDCl_3$): δ -53.4 (s). ^1H NMR (400.1 MHz, $CDCl_3$): δ 0.91 (t, *J* = 7.4 Hz, 6 H, CH_2C*H_3*), 2.16 (t, ^2J(PH) = 4.6 Hz, 6 H, P*Me*Ph_2), 3.91 (q, *J* = 7.5 Hz, 4 H, C*H_2*CH_3), 7.29-7.34 (m, 12 H, *Ph*), 7.52-7.54 (m, 8 H, *Ph*). $^{13}C\{^1H\}$ NMR (100.6 MHz, $CDCl_3$): δ 229.9 (t, ^2J(PC) = 7.0 Hz, Re-*C*), 136.3 (t, *J* = 23.5 Hz, *Ph*), 132.9 (t, *J* = 4.3 Hz, *Ph*), 130.2, 128.1 (t, *J* = 4.6 Hz, *Ph*), 31.5 (s, *C*$H_2$$CH_3$), 14.9 (s, CH_2*C*H_3), 12.7 (t, ^1J (PC) = 18.6 Hz, P*Me*Ph_2). Anal. Calcd. for $C_{32}H_{36}Cl_3P_2Re$: C, 49.58; H, 4.68. Found: C, 49.80; H,4.70.

2. Computational studies.

Computational details. The optimizations were performed with the Gaussian 16 software package [3] at the B3LYP level of density functional theory (DFT). [4] DFT/GENECP level had been done by implementing def2-TZVP basis set for Re atom. [5] The 6-311G(2d,p) basis set had been used for the rest of atoms. [6] Nucleus-independent chemical shift (NICS) values were calculated at the B3LYP-GIAO//6-311G(2d,p)/def2-TZVP level. [7] The anisotropy of the current density was calculated with the AICD 2.0 program computing the NMR properties using the CSGT method with the geometries previously obtained for **8a**. [8] GIMIC analysis was finished by GIMIC code [9] based on the formatted check point file of Gaussian and rendered by ParaView visualization program. [10] Current densities were obtained from the wavefunction for GIAO NMR computations for a magnetic field applied perpendicular to the ring plane of the molecules within the context of the Quantum Theory of Atoms in Molecules using AIMAll. [11] The molecular orbital composition was analyzed using Multiwfn, [12] a multifunctional wavefunction analyzer.

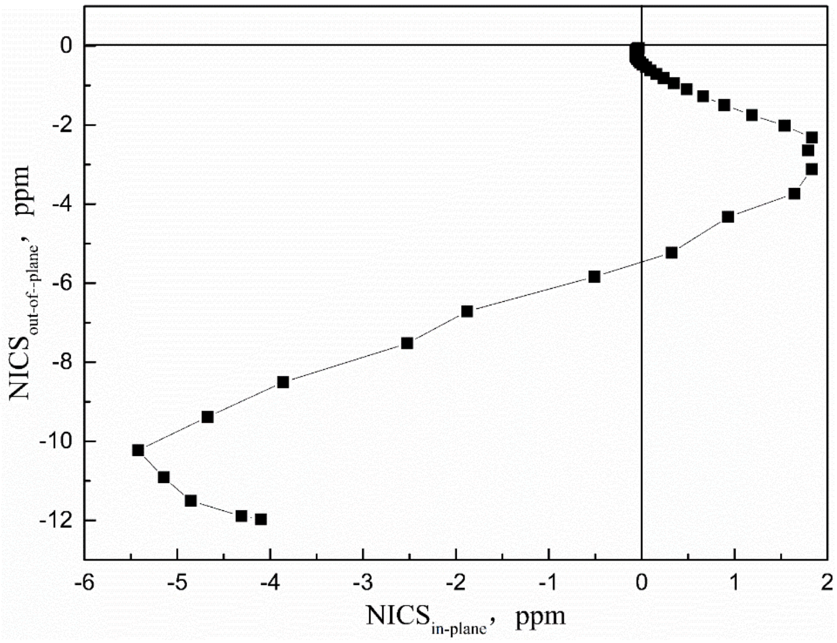

Figure S1. NICS$_{in-out}$ curve for **8a** with AIM processing.

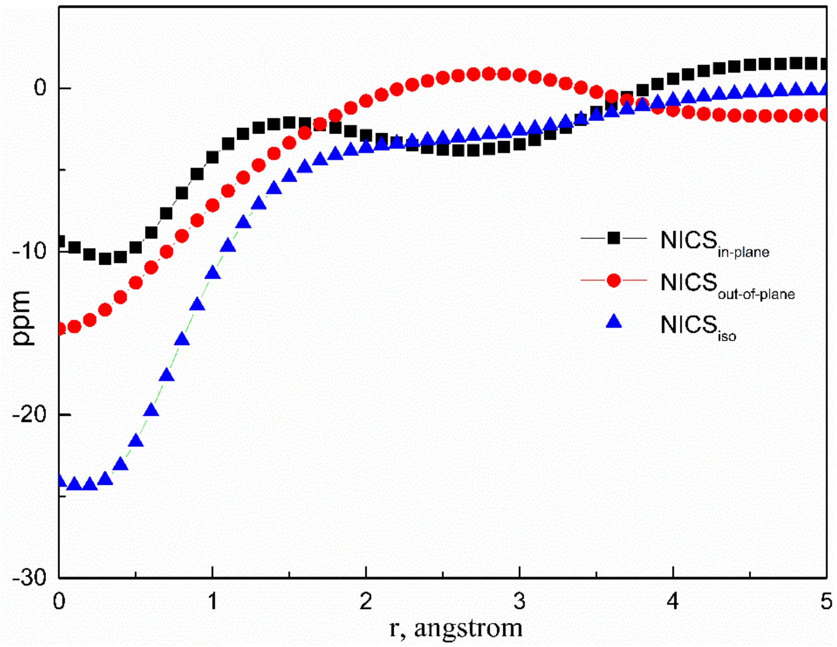

Figure S2. NICS-scan curves for **8a** without AIM processing.

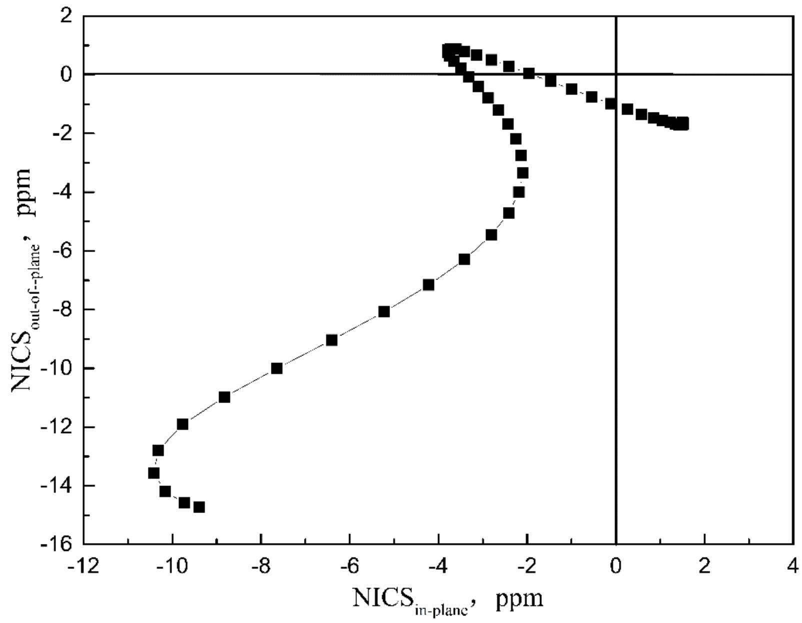

Figure S3. $NICS_{in\text{-}out}$ curve for **8a** without AIM processing.

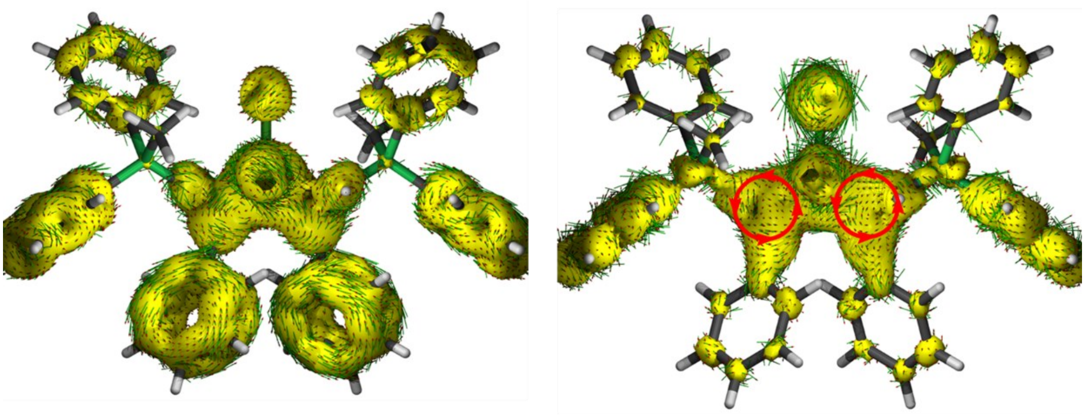

π σ

Figure S4. AICD plots of **8a** separated into the π contributions (left) and σ contributions (right) with an isosurface value of 0.03. For AICD maps, the magnetic field vector is orthogonal with respect to the monocyclic ring plane and points downward (anti-clockwise currents are diatropic).

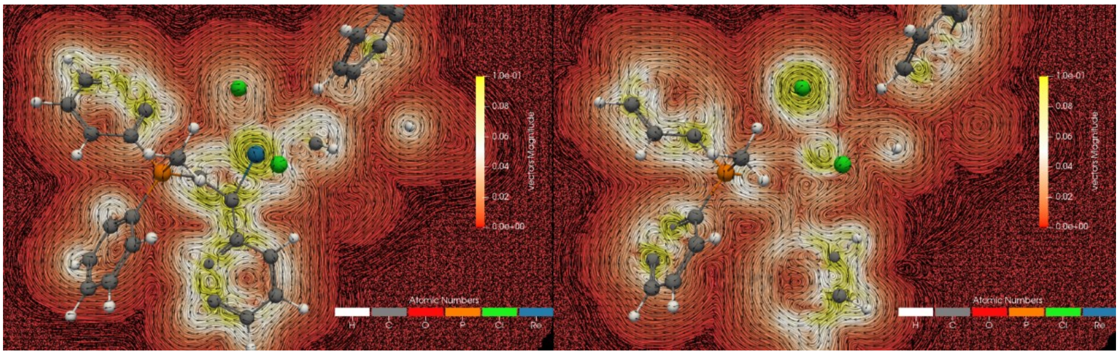

Figure S5. GIMIC maps of **8a** in the three-membered metallacycle plane (left) and 1.5 Bohr above the plane (right). The magnetic field vector is orthogonal with respect to the ring plane and points upward (clockwise currents are diatropic).

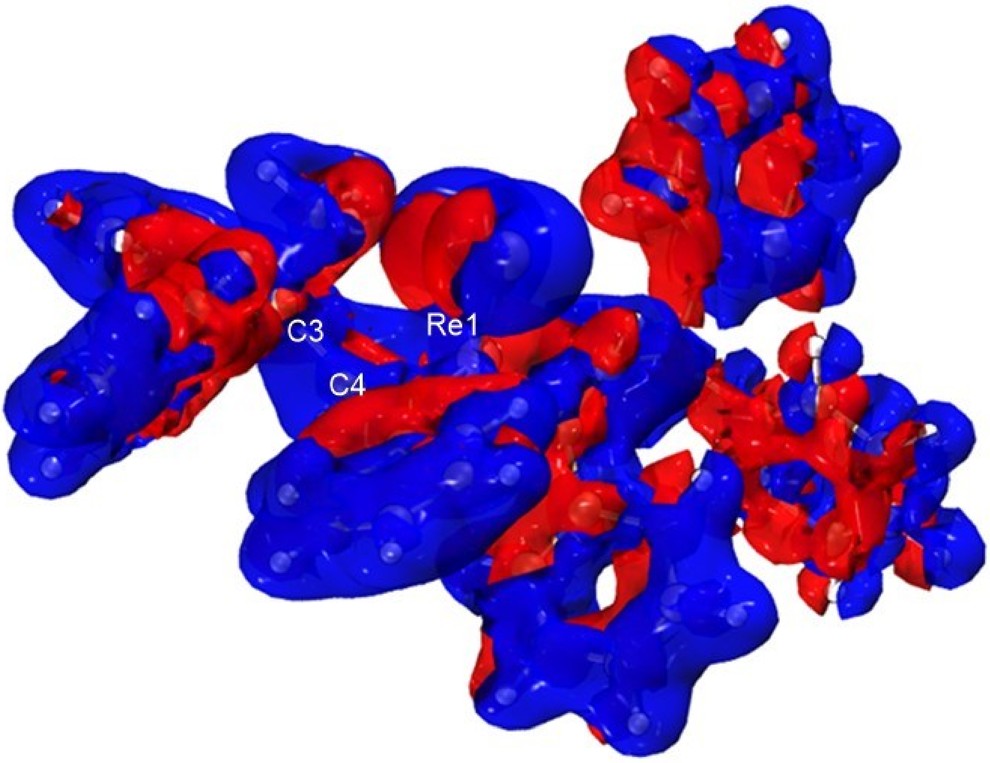

Figure S6. GIMIC Jmod plots for **8a**, isovalue = 0.02 a.u.

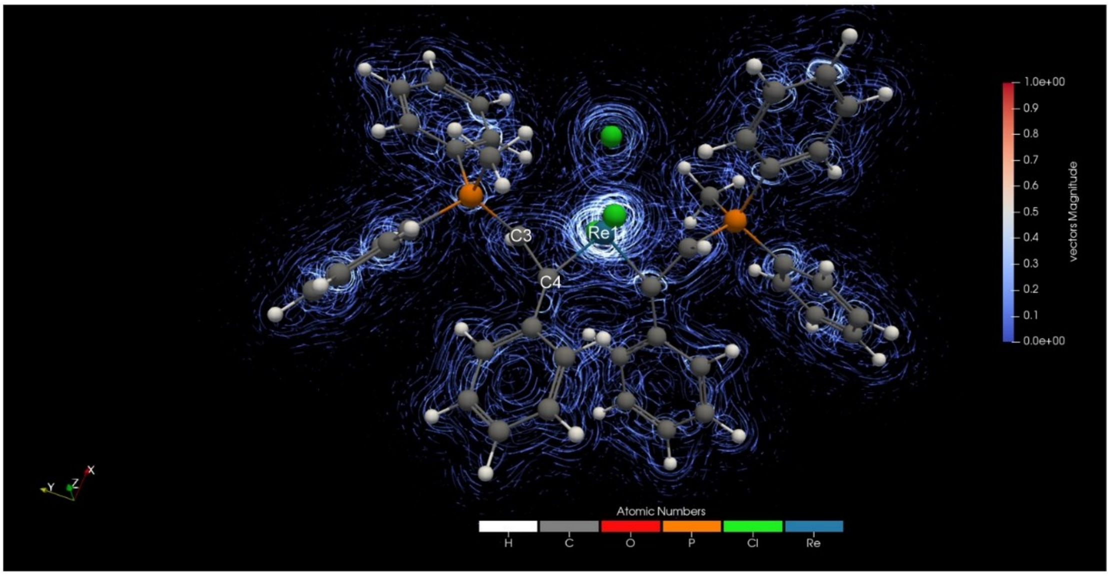

Figure S7. GIMIC streamline plots of the current densities for single metallacycle cycle of **8a**.

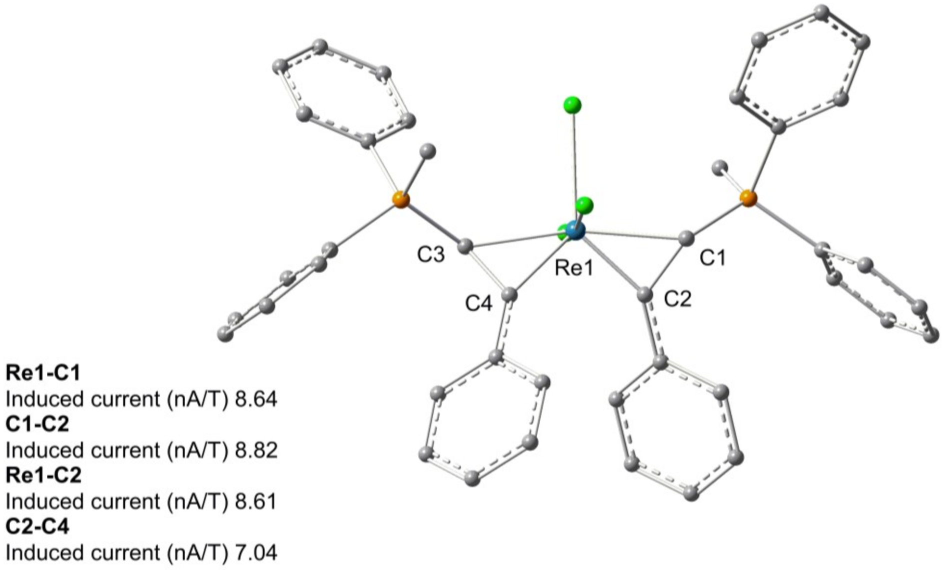

Figure S8. The magnetically induced currents intensities passing through each bond in nA/T. The positive values represent diamagnetic currents. In the calculation of the current intensity on the three-membered metallacycle, the direction of the magnetic field is perpendicular to the plane of the three-membered ring. When calculating the current intensity between the two non-coplanar rings, the magnetic field is perpendicular to the plane formed by the three atoms of Re1, C2, and C4.

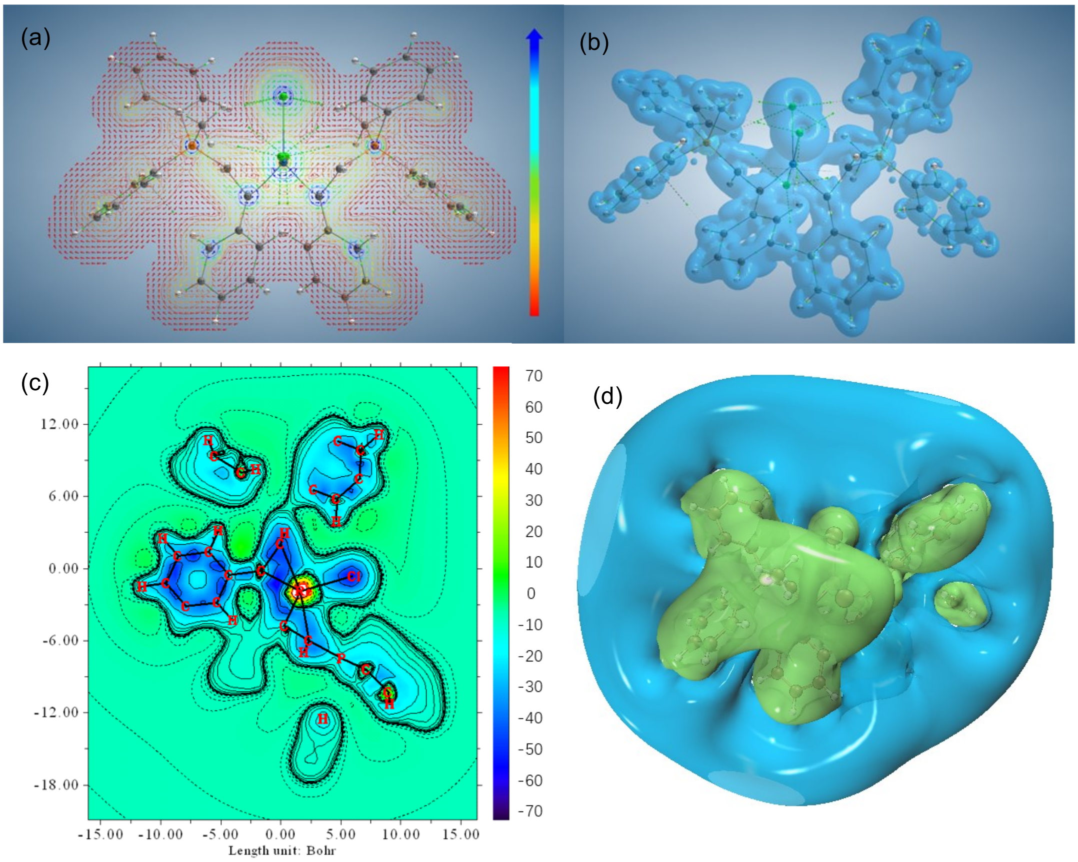

Figure S9. (a) Current intensity plotted on the mean planes of **8a**. Red to blue colors represent 0 to 0.002 atomic units of current density. (b) Isosurface of current density at J = 0.0003 a.u. for a magnetic field applied perpendicular to the plane formed by the three atoms of Re1, C2, and C4. (c) NICS-grid for **8a**. (d) ICSS plots for **8a**. Green and blue correspond to $ICSS_{ZZ}$ = 5.0 (shielding) and −2.5 ppm (deshielding), respectively.

3. X-ray crystallographic study of complexes 3a, 8a and 9.

Crystals were grown in DCM/*n*-hexane. All the crystals were mounted on the glass fibers. The diffraction intensity data of **3a** (CCDC no. 2205733), **8a** (CCDC no. 2205745) and **9** (CCDC no. 2205743) were collected on a Rigaku SuperNova, Dual, Atlas diffractometer, using monochromatized Cu-Kα radiation (λ = 1.54184 Å). Lattice determination, data collection and reduction were carried out using CrysAlisPro software (version 1.171.35.19). Empirical absorption corrections were performed using spherical harmonics, implemented in SCALE3 ABSPACK scaling algorithm in the CrysAlisPro software suite. Structure solution and refinement for all compounds were performed using the Olex2 software package. [13] All the structures were solved with the SHELXT [14] structure solution program using Intrinsic Phasing and refined with the SHELXL [15] refinement package using Least Squares minimization. All non-hydrogen atoms were refined anisotropically with a riding model for the hydrogen atoms. The crystal data are listed in Table S1. Crystallographic data have been deposited with the Cambridge Crystallographic Data Centre.

Table S1. Crystallographic data and refinement details for **3a**, **8a** and **9**.

	Complex **3a**	Complex **8a**	Complex **9**
Empirical formula	$C_{54}H_{46}Cl_7P_2Re$	$C_{42}H_{38}Cl_3P_2Re$	$C_{32}H_{36}P_2Cl_3Re$
Formula weight	1191.20	897.21	775.10
Temperature/K	173.01(10)	220.02(10)	100.01(10)
Crystal system	triclinic	orthorhombic	monoclinic
Space group	P-1	$Pna2_1$	C2/c
a/Å	10.9390(6)	23.4928(3)	11.0820(2)
b/Å	13.1090(6)	11.58031(15)	18.4374(3)
c/Å	18.4122(10)	13.76598(19)	15.6667(3)
α/°	85.943(4)	90	90
β/°	80.643(4)	90	107.276(2)
γ/°	72.033(4)	90	90
Volume/Å^3	2477.5(2)	3745.08(9)	3056.66(10)
Z	2	4	4
ρ_{calc}g/cm^3	1.597	1.591	1.684
μ/mm^{-1}	9.164	9.344	11.330
F(000)	1188.0	1784.0	1536.0
Crystal size/mm^3	0.08 × 0.06 × 0.05	0.15 × 0.12 × 0.03	0.08 × 0.06 × 0.05
Radiation	CuKα (λ = 1.54184)	CuKα (λ = 1.54184)	CuKα (λ = 1.54184)
2Θ range for data	4.866 to 154.378	8.512 to 151.238	9.594 to 151.082
Index ranges	$-13 \le h \le 13$, $-14 \le k \le 16$, $-17 \le l \le 22$	$-18 \le h \le 29$, $-13 \le k \le 13$, $-16 \le l \le 9$	$-13 \le h \le 13$, $-20 \le k \le 23$, $-18 \le l \le 19$
Reflections collected	16048	8207	8945

Independent reflections	10015 [R_{int} = 0.0312, R_{sigma} = 0.0447]	4739 [R_{int} = 0.0393, R_{sigma} = 0.0519]	3107 [R_{int} = 0.0215, R_{sigma} = 0.0231]
Data/restraints/parameters	10015/0/577	4739/7/435	3107/0/175
Goodness-of-fit on F^2	1.025	1.002	1.041
Final R indexes [I>=2σ (I)]	R_1 = 0.0283, wR_2 = 0.0678	R_1 = 0.0357, wR_2 = 0.0952	R_1 = 0.0167, wR_2 = 0.0417
Final R indexes [all data]	R_1 = 0.0324, wR_2 = 0.0699	R_1 = 0.0394, wR_2 = 0.0996	R_1 = 0.0174, wR_2 = 0.0421
Largest diff. peak/hole / e $Å^{-3}$	0.70/-1.09	2.14/-1.43	0.67/-0.51

4. NMR spectra

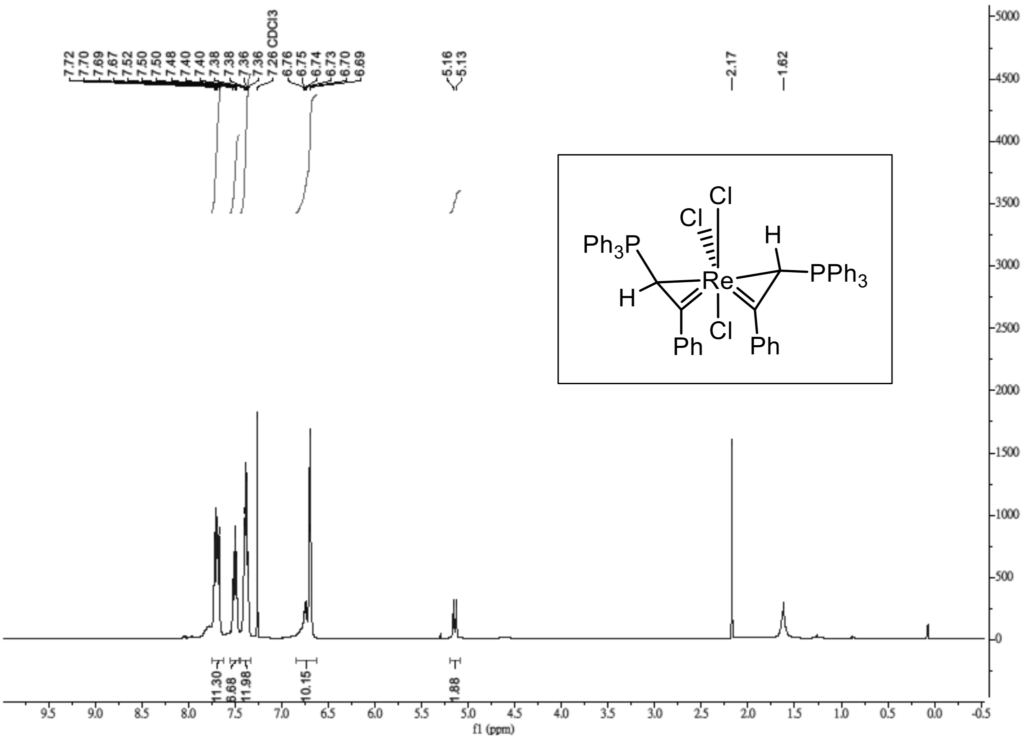

Figure S10. The ^{1}H NMR spectrum of complex **3a** in $CDCl_3$ at 400.1 MHz.

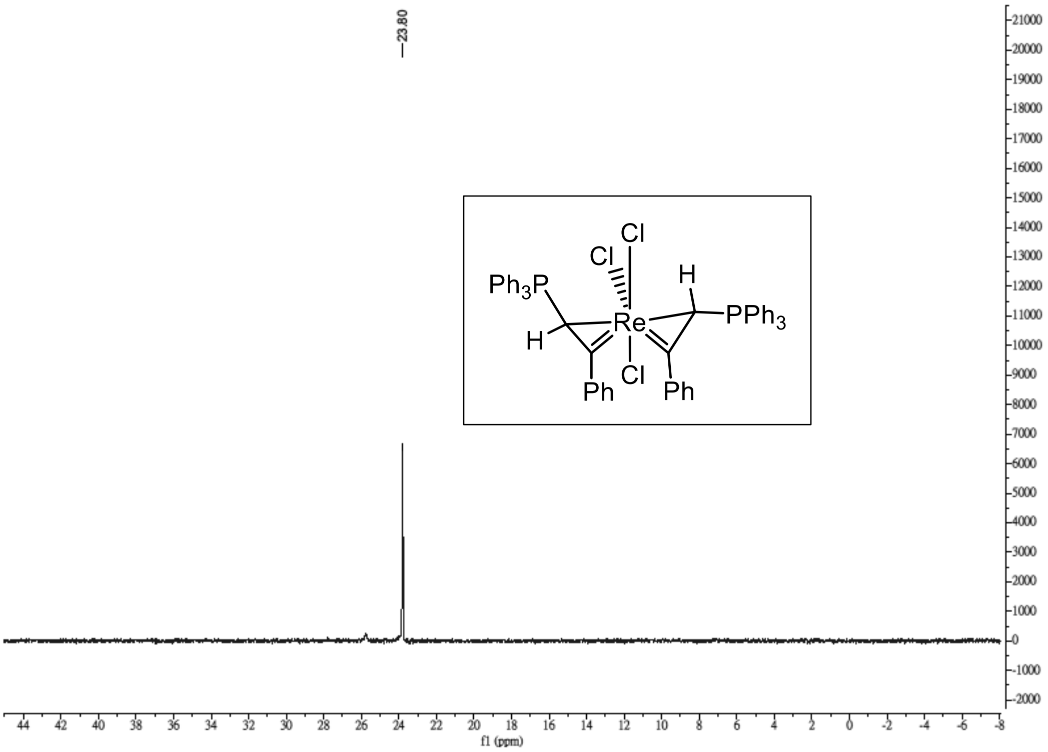

Figure S11. The $^{31}P\{^{1}H\}$ NMR spectrum of complex **3a** in $CDCl_3$ at 162.0 MHz.

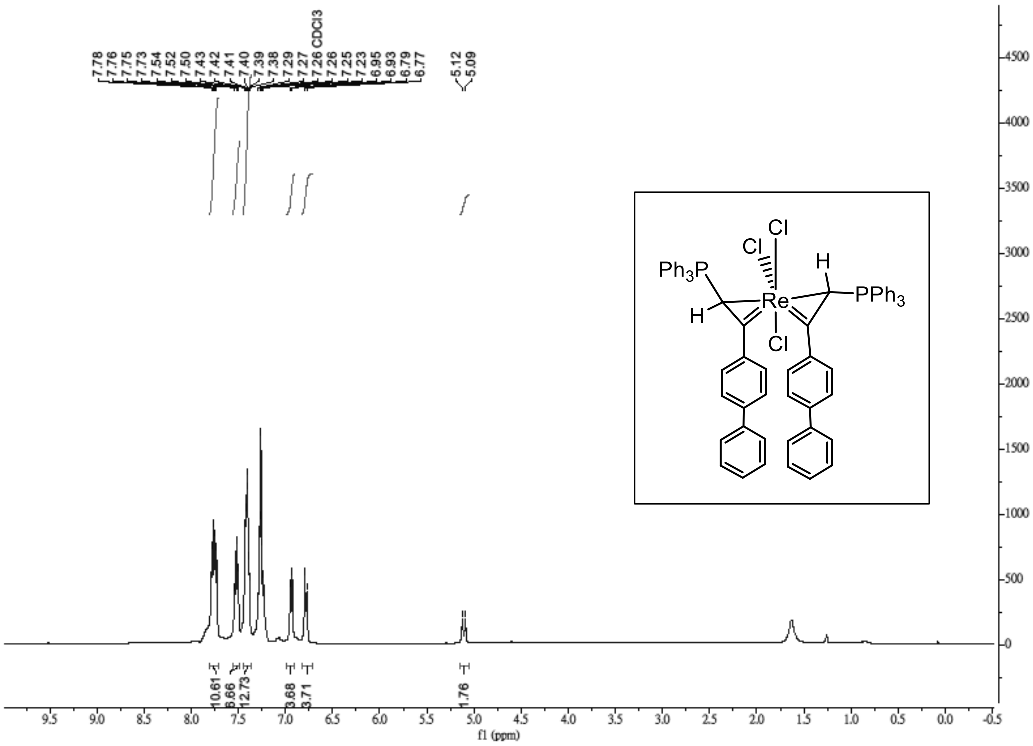

Figure S12. The ^{1}H NMR spectrum of complex **3b** in $CDCl_3$ at 400.1 MHz.

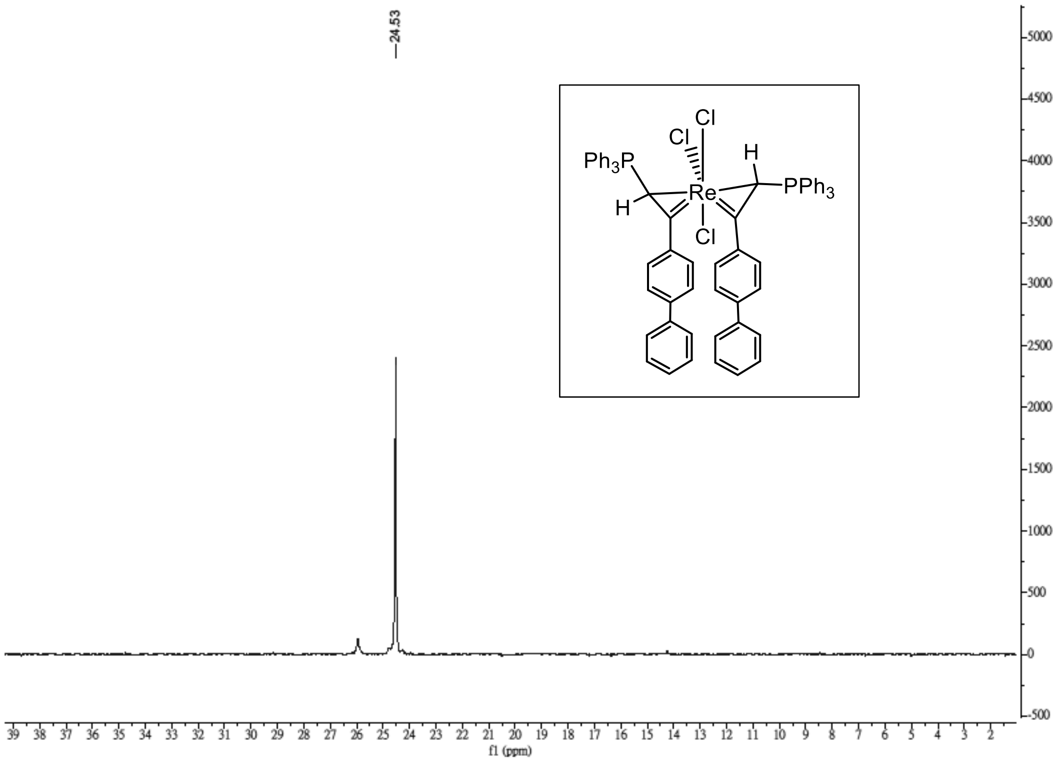

Figure S13. The $^{31}P\{^1H\}$ NMR spectrum of complex **3b** in $CDCl_3$ at 162.0 MHz.

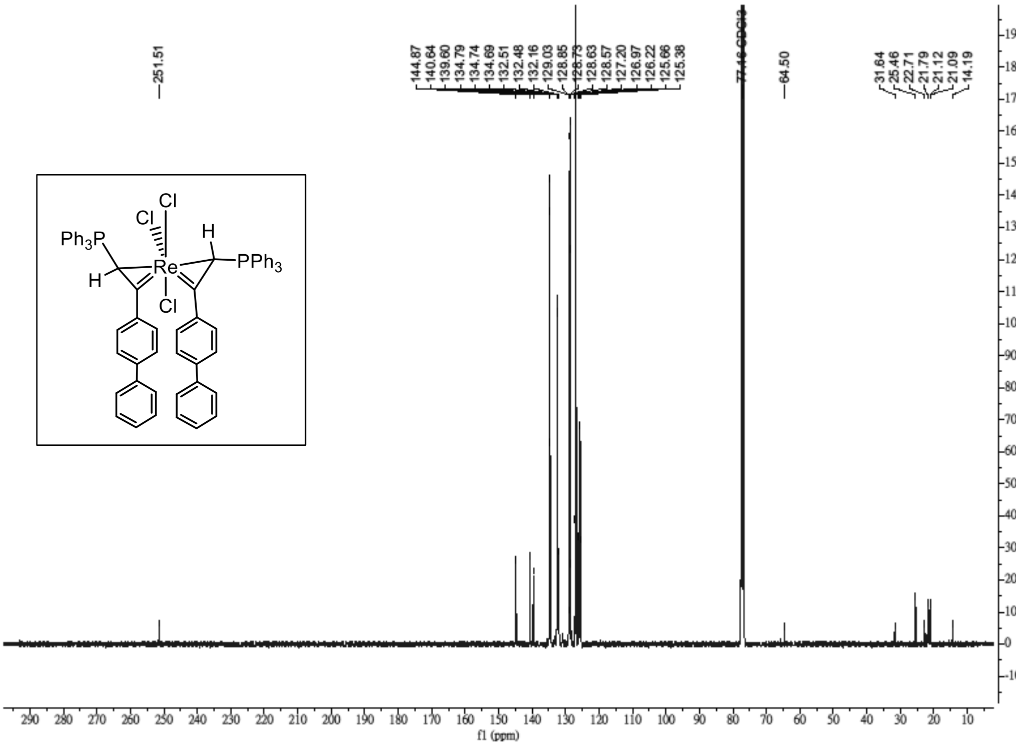

Figure S14. The $^{13}C\{^{1}H\}$ NMR spectrum of complex **3b** in $CDCl_3$ at 100.6 MHz.

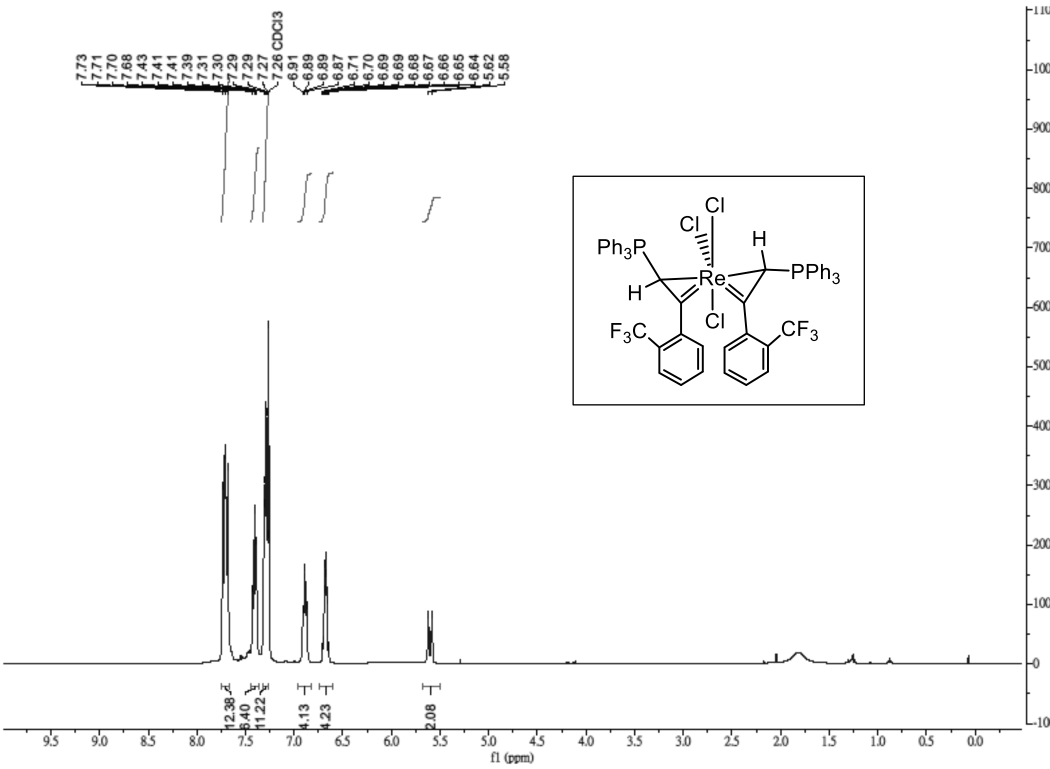

Figure S15. The ^{1}H NMR spectrum of complex **3c** in $CDCl_3$ at 400.1 MHz.

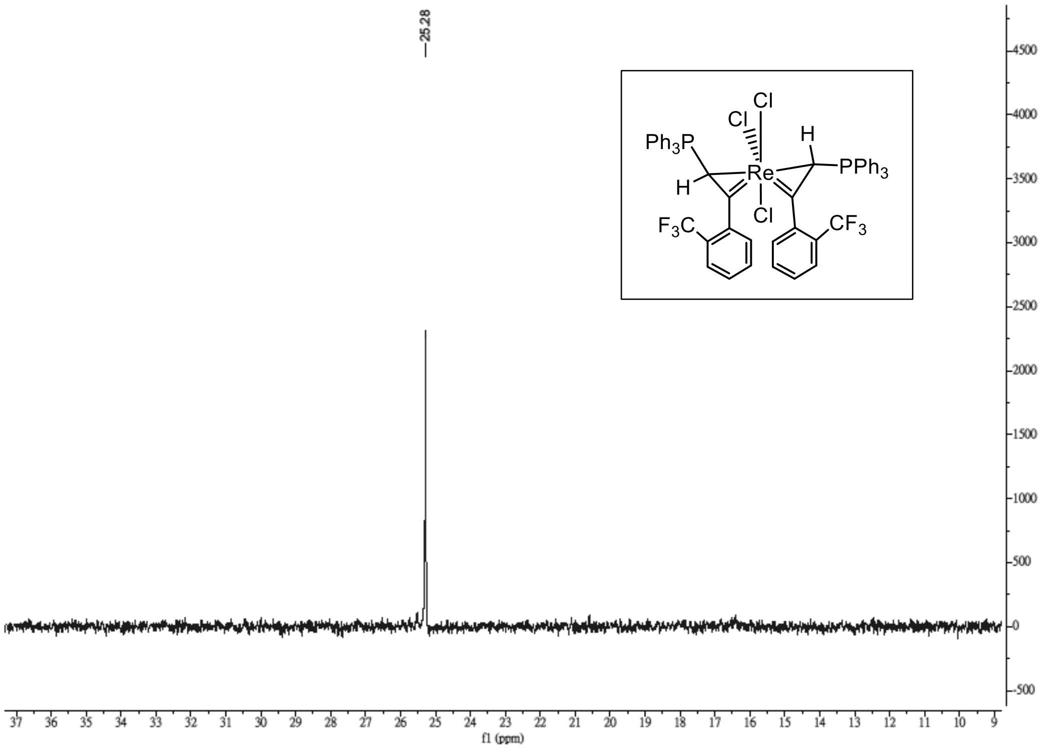

Figure S16. The $^{31}P\{^1H\}$ NMR spectrum of complex **3c** in $CDCl_3$ at 162.0 MHz.

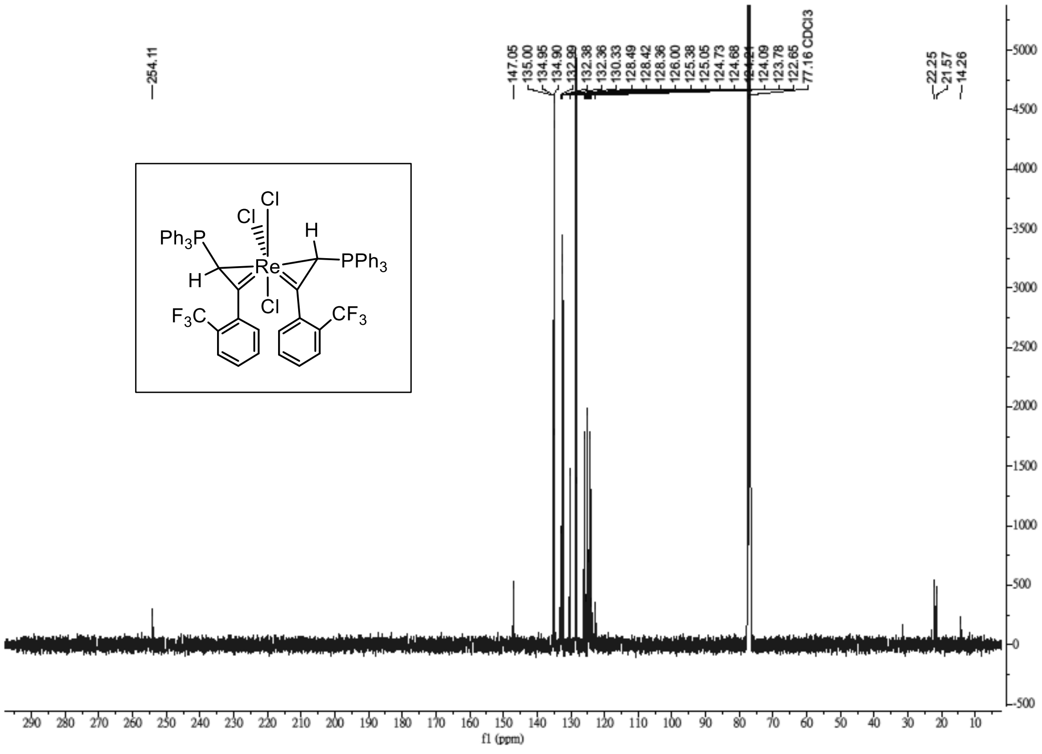

Figure S17. The $^{13}C\{^1H\}$ NMR spectrum of complex **3c** in $CDCl_3$ at 100.6 MHz.

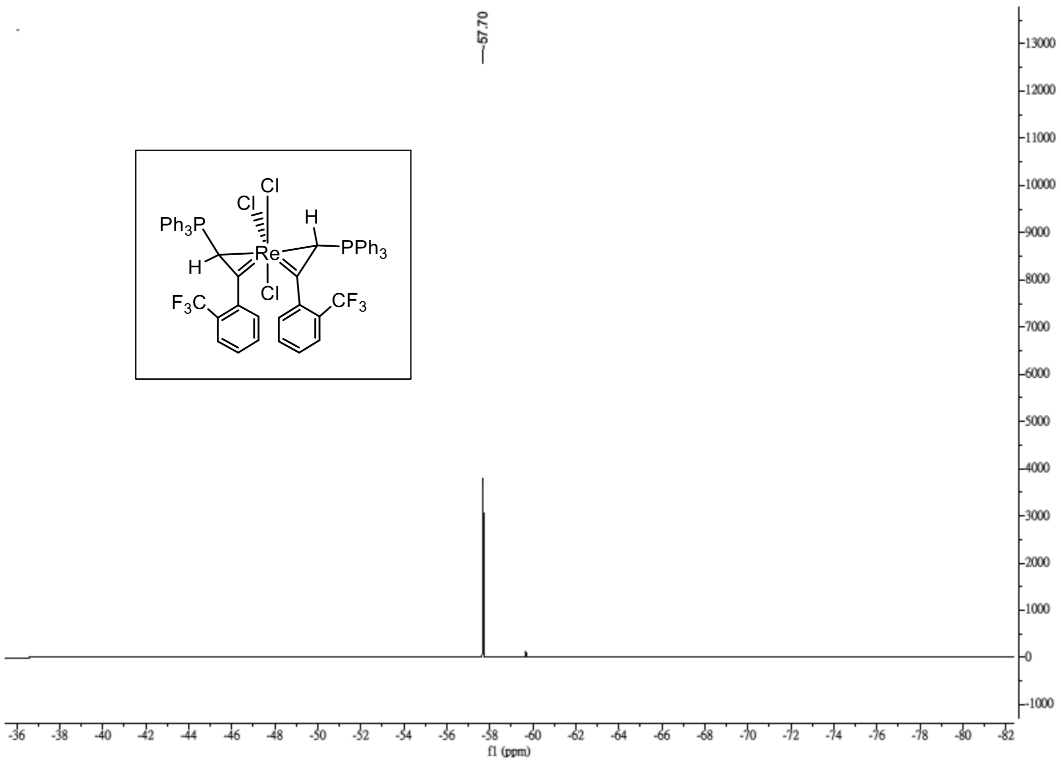

Figure S18. The ^{19}F NMR spectrum of complex **3c** in $CDCl_3$ at 376 MHz.

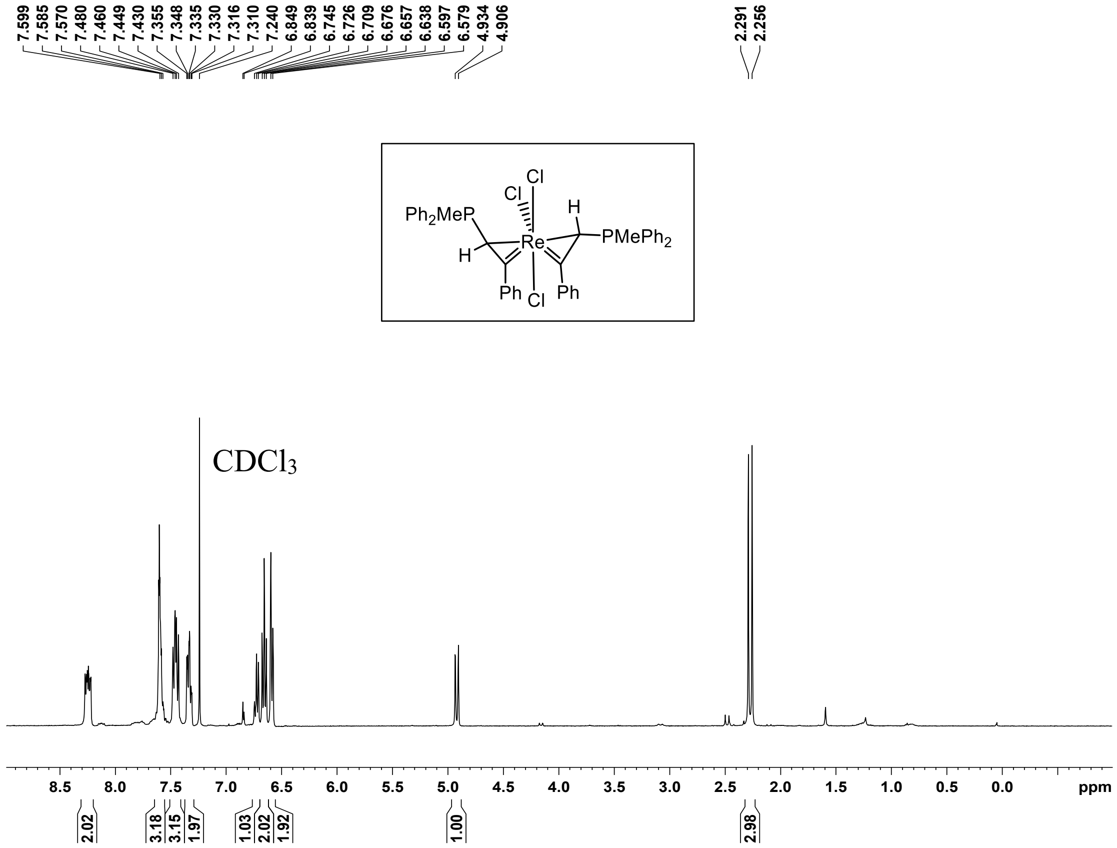

Figure S19. The ^{1}H NMR spectrum of complex **8a** in $CDCl_3$ at 400.1 MHz.

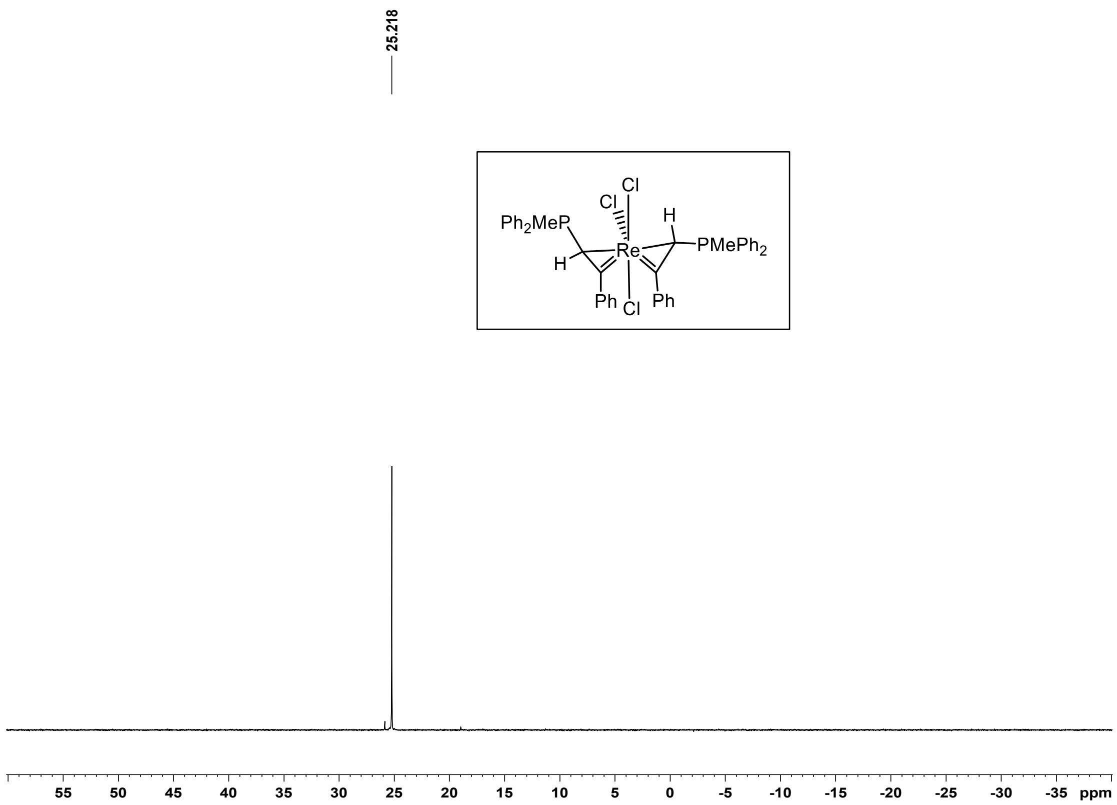

Figure S20. The $^{31}P\{^{1}H\}$ NMR spectrum of complex **8a** in $CDCl_3$ at 162.0 MHz.

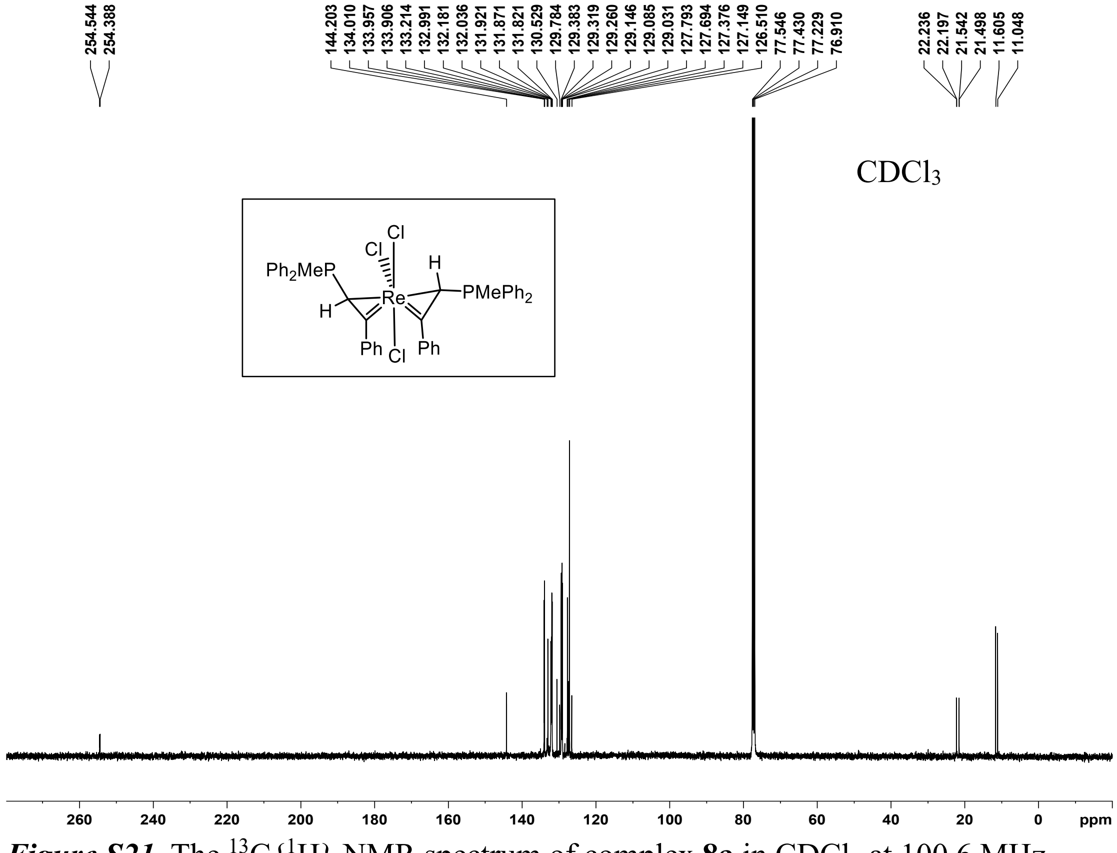

Figure S21. The $^{13}C\{^{1}H\}$ NMR spectrum of complex **8a** in $CDCl_3$ at 100.6 MHz.

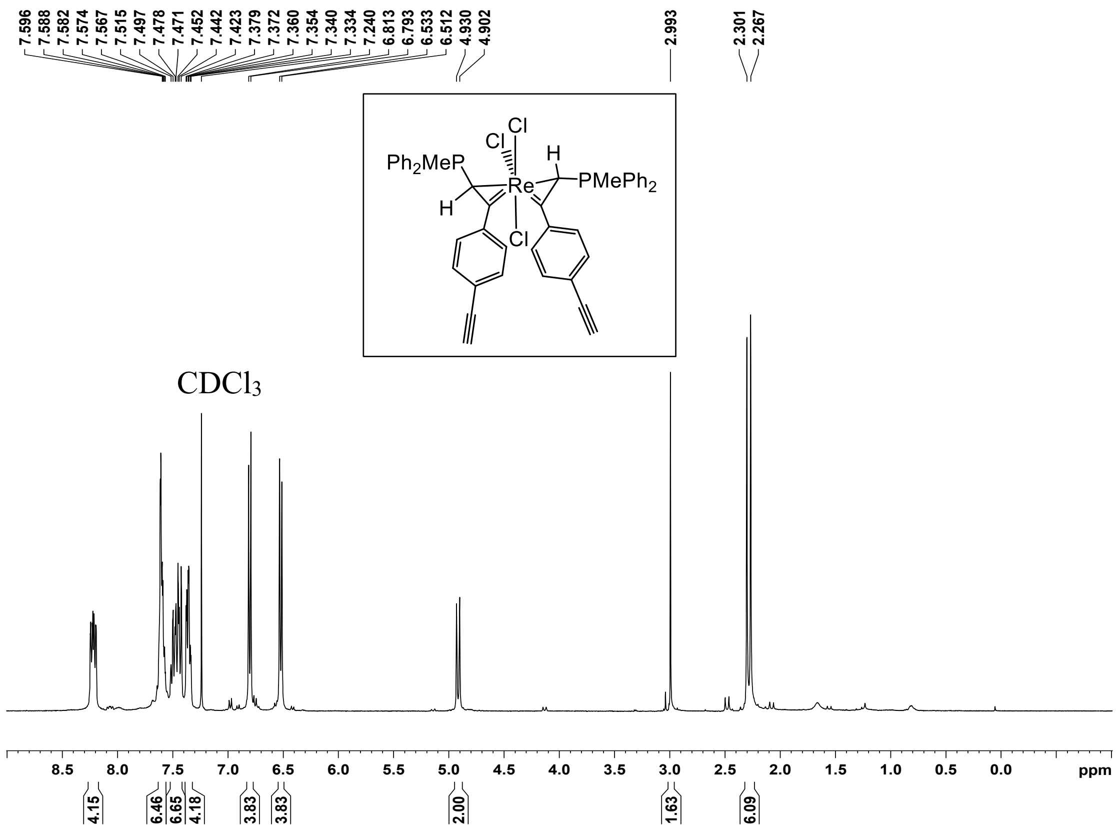

Figure S22. The ^{1}H NMR spectrum of complex **8b** in $CDCl_3$ at 400.1 MHz.

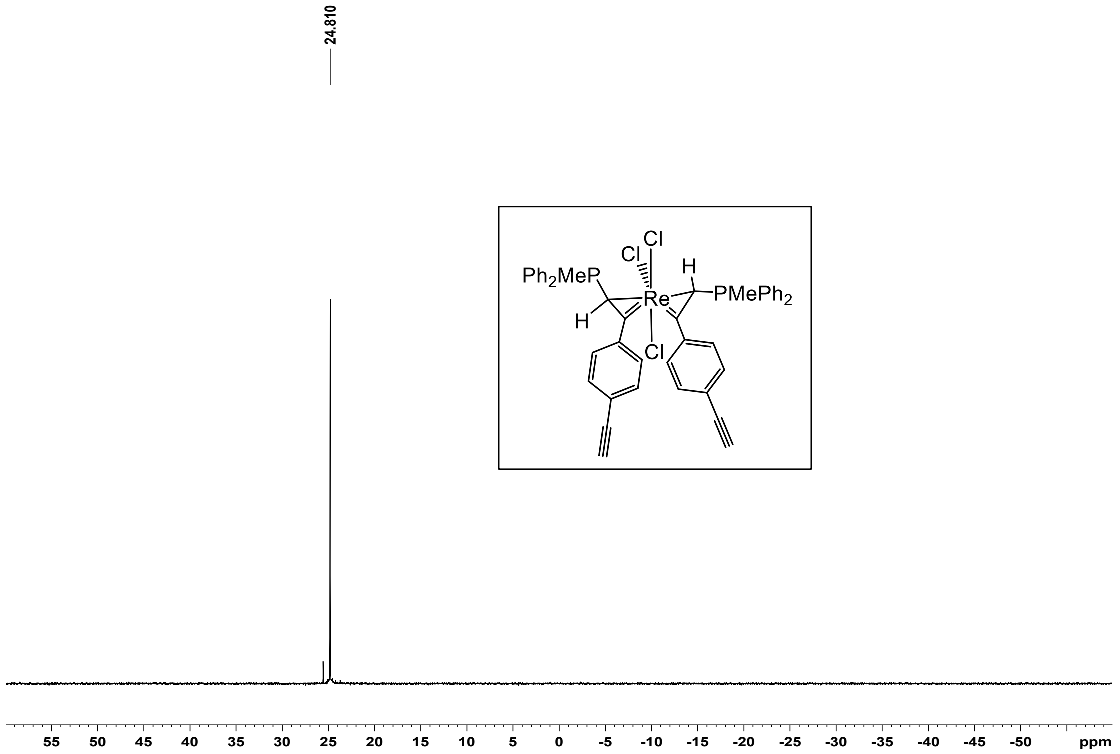

Figure S23. The $^{31}P\{^1H\}$ NMR spectrum of complex **8b** in $CDCl_3$ at 162.0 MHz.

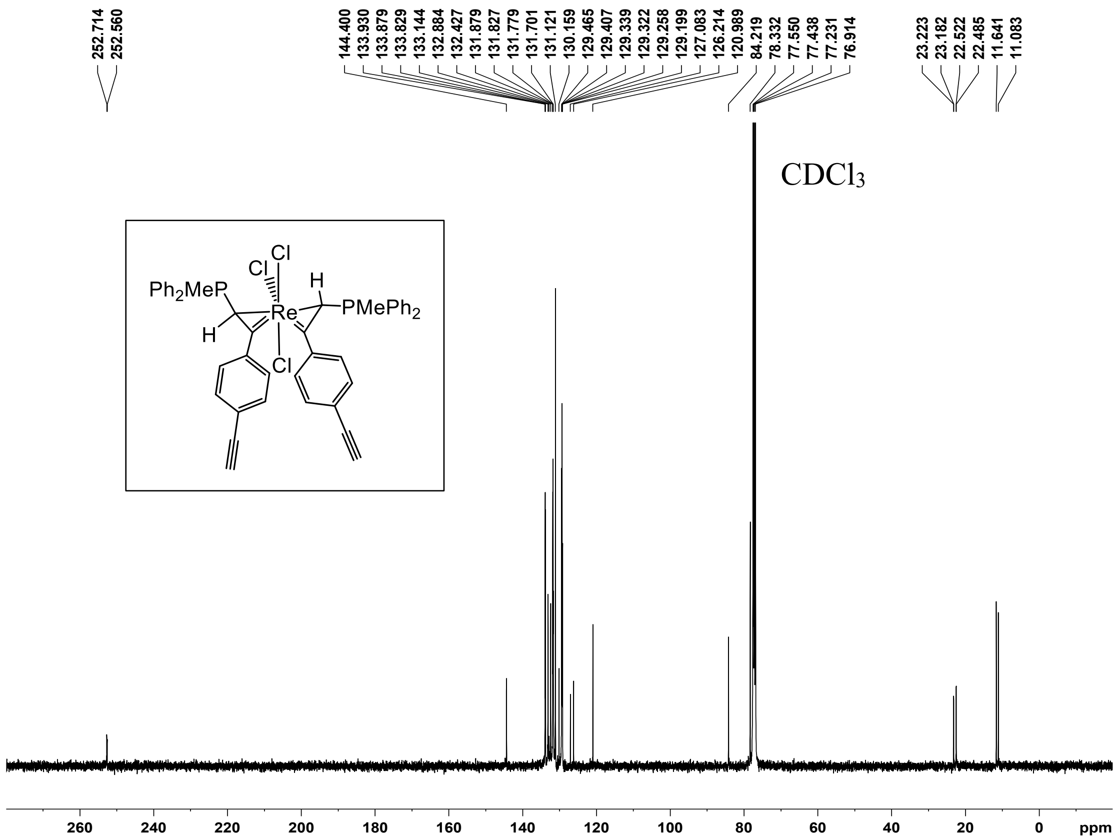

Figure S24. The $^{13}C\{^{1}H\}$ NMR spectrum of complex **8b** in $CDCl_3$ at 100.6 MHz.

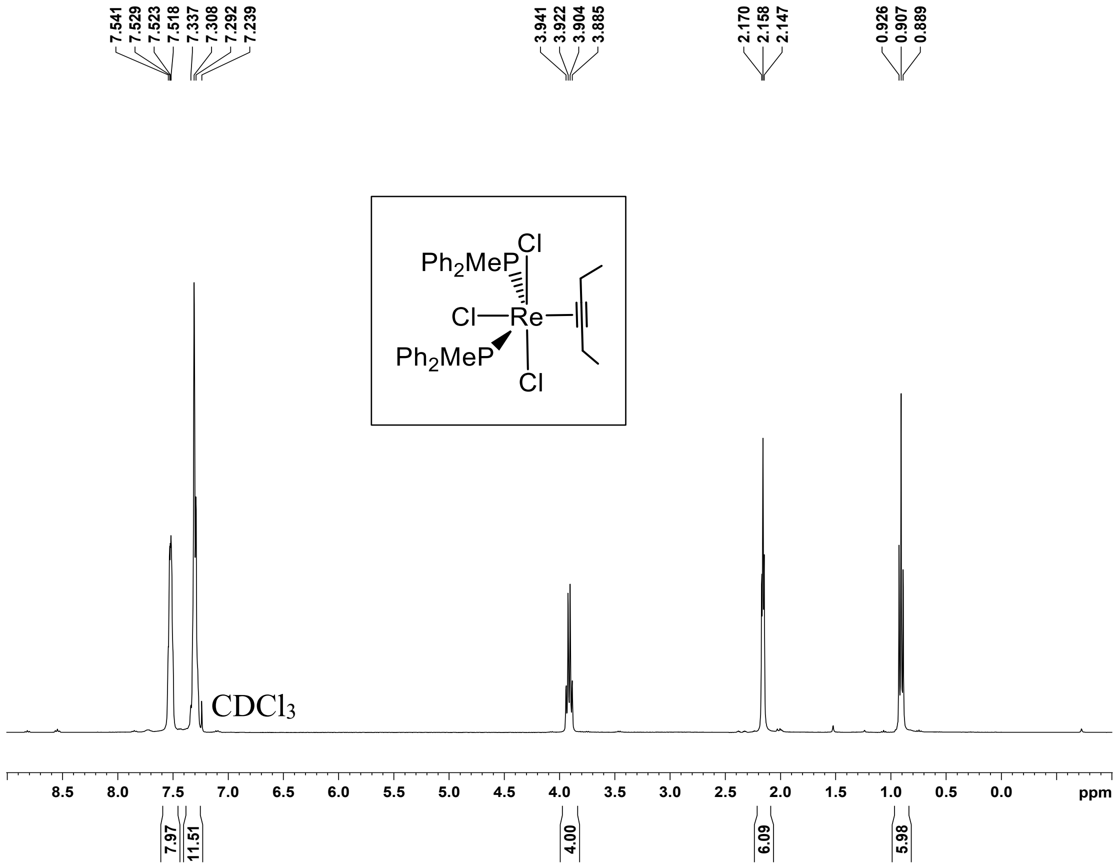

Figure S25. The ^{1}H NMR spectrum of complex **9** in $CDCl_3$ at 400.1 MHz.

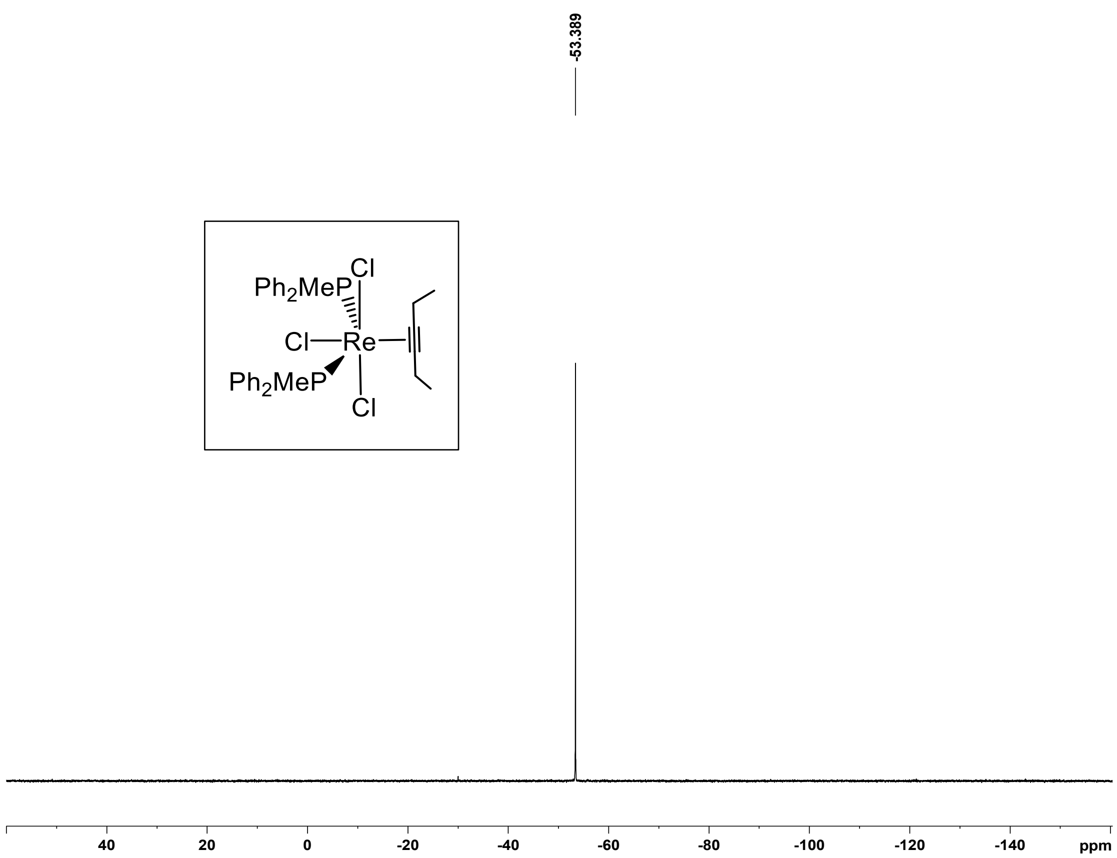

Figure S26. The $^{31}P\{^1H\}$ NMR spectrum of complex **9** in $CDCl_3$ at 162.0 MHz.

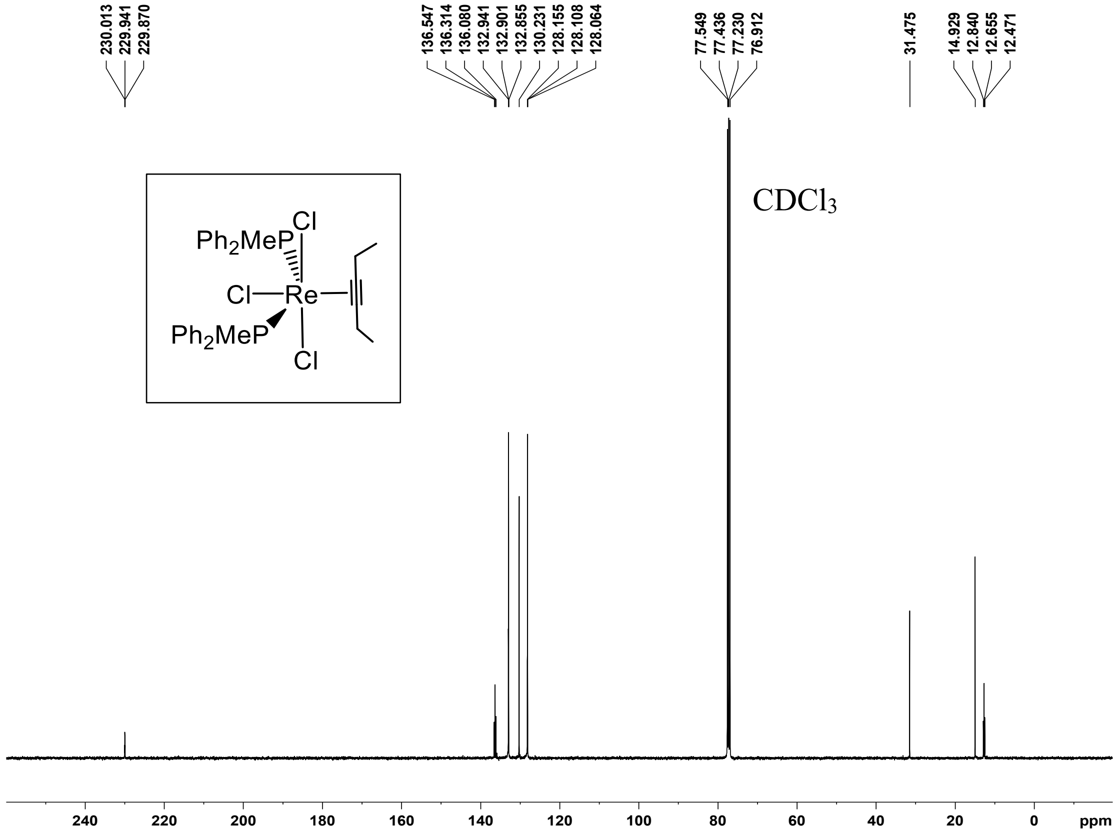

Figure S27. The $^{13}C\{^1H\}$ NMR spectrum of complex **9** in $CDCl_3$ at 100.6 MHz.

5. The Calculated Cartesian Coordinates with Electronic Energies

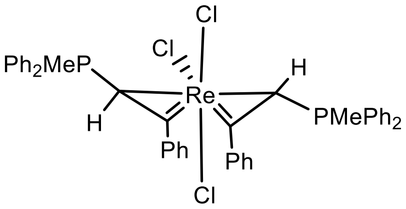

8a

E = -3765.67385826 A.U.

Re	0.87055791	0.00000000	0.00012922
Cl	1.09402770	-0.28918463	-2.44385138
Cl	3.39717606	0.00035765	0.00035734
Cl	1.09355016	0.28915606	2.44406026
P	1.50216276	3.53369879	-0.03305121
P	1.50303314	-3.53348431	0.03315555
C	2.29620529	3.28374771	1.57430062
H	2.82611989	4.19435069	1.85564259
H	2.99563208	2.45434477	1.46498271
H	1.56578973	3.00521688	2.33133355
C	2.29765668	-3.28326097	-1.57386998
H	1.56748419	-3.00498762	-2.33123499
H	2.82797413	-4.19371025	-1.85494256
H	2.99675496	-2.45362136	-1.46425545
C	0.62418166	2.12533538	-0.65188302
H	0.59199809	2.19490747	-1.74154156
C	-0.43538852	1.38034315	0.00010158
C	-1.74470547	1.76767521	0.47859249
C	-2.42031355	2.86692205	-0.07993169
H	-1.94596846	3.42994736	-0.87347971
C	-3.68691413	3.22090013	0.35954422
H	-4.19794408	4.06293597	-0.09285188
C	-4.29647426	2.50317717	1.38473510
H	-5.28255752	2.78580404	1.73486943
C	-3.63234209	1.42370722	1.96123320
H	-4.09965989	0.86731500	2.76538322
C	-2.37543841	1.05187313	1.51042946
H	-1.85072882	0.22175491	1.96233472
C	0.62455014	-2.12539087	0.65193347
H	0.59212973	-2.19516938	1.74157226
C	-0.43509782	-1.38060715	-0.00016132
C	-1.74429506	-1.76817433	-0.47877678
C	-2.37510420	-1.05245104	-1.51062183
H	-1.85051273	-0.22222080	-1.96246108
C	-3.63190671	-1.42450393	-1.96151716
H	-4.09928797	-0.86816663	-2.76566844

C	-4.29586162	-2.50413415	-1.38511232
H	-5.28187249	-2.78693046	-1.73531157
C	-3.68621766	-3.22179750	-0.35993098
H	-4.19710276	-4.06396720	0.09237645
C	-2.41971305	-2.86759202	0.07964171
H	-1.94530007	-3.43057160	0.87318209
C	2.81781218	3.93924854	-1.22105585
C	3.32626046	2.95296436	-2.07007058
H	2.92517866	1.94847400	-2.05167601
C	4.37430281	3.26019352	-2.93135570
H	4.76293144	2.48979522	-3.58572114
C	4.92279020	4.53614349	-2.94639596
H	5.74036790	4.76788688	-3.61902112
C	4.42444924	5.51804545	-2.09506866
H	4.85079198	6.51404038	-2.10130085
C	3.37562466	5.22283678	-1.23595248
H	2.98736192	5.99629336	-0.58449513
C	0.47233593	5.03934031	0.11041315
C	0.02782840	5.67829678	-1.05267296
H	0.34989484	5.32230369	-2.02410461
C	-0.81884544	6.77451031	-0.97218885
H	-1.15192873	7.26401970	-1.87952210
C	-1.23730906	7.24392811	0.26989086
H	-1.89901080	8.09959719	0.33190399
C	-0.80464509	6.61270072	1.42890060
H	-1.12984457	6.97073966	2.39816154
C	0.04604265	5.51507185	1.35154974
H	0.36590181	5.03072538	2.26475384
C	0.47353887	-5.03929473	-0.11083171
C	0.04741906	-5.51466973	-1.35216829
H	0.36723588	-5.02993608	-2.26518584
C	-0.80306530	-6.61242623	-1.42996237
H	-1.12813189	-6.97017344	-2.39937333
C	-1.23568532	-7.24414872	-0.27120862
H	-1.89721264	-8.09992734	-0.33356343
C	-0.81739484	-6.77509050	0.97106779
H	-1.15045754	-7.26497883	1.87820422
C	0.02906840	-5.67874828	1.05199425
H	0.35099405	-5.32303020	2.02356893
C	2.81841189	-3.93885043	1.22152487
C	3.37646547	-5.22233025	1.23656250
H	2.98848416	-5.99587409	0.58504366
C	4.42516302	-5.51732090	2.09591140
H	4.85168834	-6.51323757	2.10226142

C	4.92314912	-4.53530230	2.94731122
H	5.74063550	-4.76687539	3.62010386
C	4.37442945	-3.25945427	2.93212222
H	4.76278187	-2.48896400	3.58654384
C	3.32650467	-2.95244618	2.07061381
H	2.92523742	-1.94803314	2.05211828

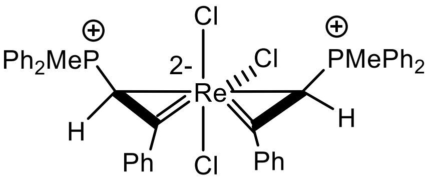

8a'

E = -3765.665668 A.U.

Re	0.01849800	-0.59978000	-0.24229000
Cl	0.29173500	-1.75056200	-2.37575400
Cl	0.36655600	-2.88529100	0.77333800
Cl	-0.30234000	0.08322500	2.12171500
P	3.51619600	-0.53618000	0.57883400
P	-3.31183100	-0.90947600	0.91402600
C	2.97928500	-0.73094200	2.29634300
H	3.84300100	-0.95867200	2.92159900
H	2.26415400	-1.55415000	2.31854800
H	2.47025100	0.16565900	2.64597200
C	-2.89248200	-2.24027900	2.07486100
H	-2.70017800	-3.13772200	1.48723100
H	-3.71773900	-2.42327000	2.76304100
H	-1.97363900	-1.98211600	2.59781100
C	2.19656400	-0.16622100	-0.54674400
H	2.49913800	-0.46965000	-1.55095800
C	1.20735600	0.88372300	-0.42423000
C	1.33313800	2.32151600	-0.30956600
C	2.46298700	2.99096700	-0.81279600
H	3.24951900	2.41634200	-1.28470600
C	2.56869300	4.37162400	-0.72972300
H	3.44174200	4.86949300	-1.13558800
C	1.56205200	5.11426100	-0.11963900
H	1.64906200	6.19215300	-0.04611200
C	0.44431000	4.46464100	0.39850400
H	-0.33835000	5.03766500	0.88233700
C	0.32288800	3.08763400	0.29842200
H	-0.53840100	2.57821900	0.70711700
C	-2.15641400	-0.84543000	-0.43202800
H	-2.43819700	-1.63555800	-1.13373700

C	-1.47139300	0.30034800	-1.01592000
C	-1.93009500	1.18715100	-2.06520200
C	-1.03878600	1.99359900	-2.79419500
H	0.01387100	1.95136400	-2.56005700
C	-1.49185500	2.81484500	-3.81334500
H	-0.78473500	3.42501100	-4.36300500
C	-2.84511200	2.85229100	-4.14136400
H	-3.19501600	3.49478200	-4.94095700
C	-3.74164700	2.04516800	-3.44897900
H	-4.79312100	2.04902500	-3.71231100
C	-3.28932500	1.21869200	-2.43053400
H	-3.98785700	0.56755300	-1.91968600
C	4.29483600	-2.09479400	0.06100600
C	3.58826200	-3.01111700	-0.72159900
H	2.57959300	-2.79729500	-1.04857000
C	4.18065800	-4.22186900	-1.06545800
H	3.62586200	-4.92680400	-1.67203600
C	5.46269600	-4.52820500	-0.62823200
H	5.91669200	-5.47461300	-0.89808200
C	6.16551700	-3.62066300	0.15935200
H	7.16509400	-3.85633700	0.50452400
C	5.58627400	-2.40746300	0.50163800
H	6.14528900	-1.70190100	1.10439000
C	4.85965200	0.70717300	0.57589500
C	5.67614000	0.82487700	-0.55474400
H	5.55310900	0.14080100	-1.38605400
C	6.65201400	1.80914000	-0.61750900
H	7.28062100	1.88733000	-1.49645800
C	6.82405300	2.69078600	0.44622800
H	7.58664500	3.45883500	0.39671800
C	6.01633800	2.58255200	1.57094800
H	6.14348400	3.26720200	2.40076900
C	5.03753300	1.59651600	1.63686300
H	4.41145400	1.53332400	2.51715300
C	-4.94331300	-1.41411400	0.24248900
C	-5.02033500	-2.58764200	-0.51795500
H	-4.13283900	-3.17918200	-0.70875500
C	-6.23517300	-3.00740400	-1.04166200
H	-6.27926200	-3.91634200	-1.62928700
C	-7.38873100	-2.26310300	-0.81512200
H	-8.33592300	-2.59121200	-1.22622100
C	-7.32139700	-1.09895600	-0.06071000
H	-8.21586200	-0.51498800	0.12051400
C	-6.10612500	-0.67513000	0.46751600

H	-6.06817900	0.23309600	1.05419600
C	-3.58883300	0.62796900	1.83814800
C	-3.72848300	0.60454000	3.22783800
H	-3.61703500	-0.32106600	3.77567400
C	-3.98584600	1.77695500	3.92497200
H	-4.08503800	1.74771100	5.00322400
C	-4.10307200	2.98360600	3.24403800
H	-4.29797100	3.89857900	3.79072500
C	-3.96424300	3.01349800	1.86151300
H	-4.05009200	3.95008100	1.32399000
C	-3.71036200	1.84205200	1.15810600
H	-3.60248500	1.88295200	0.08329900

References

[1] W. A. Skupiński, J. C. Huffman, J. W. Bruno and K. G. Caulton, *J. Am. Chem. Soc.* 1984, **106**, 8128-8136.

[2] P. P. M. Schleker, R. Honeker, J. Klankermayer, W. Leitner, *ChemCatChem* **2013**, *5*, 1762-1764.

[3] Gaussian 16, Revision A.03, M. J. Frisch, G. W. Trucks, H. B. Schlegel, G. E. Scuseria, M. A. Robb, J. R. Cheeseman, G. Scalmani, V. Barone, G. A. Petersson, H. Nakatsuji, X. Li, M. Caricato, A. V. Marenich, J. Bloino, B. G. Janesko, R. Gomperts, B. Mennucci, H. P. Hratchian, J. V. Ortiz, A. F. Izmaylov, J. L. Sonnenberg, D. Williams-Young, F. Ding, F. Lipparini, F. Egidi, J. Goings, B. Peng, A. Petrone, T. Henderson, D. Ranasinghe, V. G. Zakrzewski, J. Gao, N. Rega, G. Zheng, W. Liang, M. Hada, M. Ehara, K. Toyota, R. Fukuda, J. Hasegawa, M. Ishida, T. Nakajima, Y. Honda, O. Kitao, H. Nakai, T. Vreven, K. Throssell, J. A. Montgomery, Jr., J. E. Peralta, F. Ogliaro, M. J. Bearpark, J. J. Heyd, E. N. Brothers, K. N. Kudin, V. N. Staroverov, T. A. Keith, R. Kobayashi, J. Normand, K. Raghavachari, A. P. Rendell, J. C. Burant, S. S. Iyengar, J. Tomasi, M. Cossi, J. M. Millam, M. Klene, C. Adamo, R. Cammi, J. W. Ochterski, R. L. Martin, K. Morokuma, O. Farkas, J. B. Foresman, and D. J. Fox, Gaussian, Inc., Wallingford CT, 2016.

[4] a) A. D. Becke, *J. Chem. Phys.* 1993, **98**, 5648-5652; b) B. Miehlich, A. Savin, H. Stoll, H. Preuss, *Chem. Phys. Lett.* 1989, **157**, 200-206; c) C. Lee, W. Yang, R. G. Parr, *Phys. Rev. B.* 1988, **37**, 785-789.

[5] a) F. Weigend, R. Ahlrichs, *Phys. Chem. Chem. Phys.* 2005, **7**, 3297–3305; b) F. Weigend, *Phys. Chem. Chem. Phys.* 2006, **8**, 1057-1065.

[6] P. J. Hay, W. R. Wadt, *J. Chem. Phys.* 1985, **82**, 299-310.

[7] a) P. v. R. Schleyer, C. Maerker, A. Dransfeld, H. Jiao, N. J. R. v. E. Hommes, *J. Am. Chem. Soc.* 1996, **118**, 6317-6318; b) Z. Chen, C. S. Wannere, C. Corminboeuf, R. Puchta, P. v. R. Schleyer, *Chem. Rev.* 2005, **105**, 3842-3888; c) H. Fallah-Bagher-Shaidaei, C. S. Wannere, C. Corminboeuf, R. Puchta, P. v. R. Schleyer, *Org. Lett.* 2006, **8**, 863-866.

[8] a) R. Herges, D. Geuenich, *J. Phys. Chem. A* 2001, **105**, 3214-3220. a) D. Geuenich, K. Hess, F. Kçhler, R. Herges, *Chem. Rev.* 2005, **105**, 3758-3772.

[9] J. W. Storer, D. J. Giesen, C. J. Cramer, and D. G. Truhlar, *J. Comput.-Aided Mol. Des.* 1995, **9**, 87-110.

[10] C. M. Breneman, and K. B. Wiberg, *J. Comput. Chem.* 1990, **11**, 361-373.

[11] T. A. Keith, AIMAll, Gristmill Software, Overland Park KS, USA, 2017.

[12] a) T. Lu, F. Chen, *Acta Chim. Sinica* 2011, **69**, 2393-2406; b) T. Lu, F. Chen, *J. Comput. Chem.* 2012, **33**, 580-592.

[13] O. V. Dolomanov, L. J. Bourhis, R. J. Gildea, J. A. K. Howard, H. J. Puschmann, *Appl. Cryst.* 2009, ***42***, 339-341.

[14] G. M. Sheldrick, *Acta Cryst.* 2015, ***A71***, 3-8.

[15] G. M. Sheldrick, *Acta Cryst.* 2015, ***C71***, 3-8.
